# Smartphone App–Based Music-Facilitated Pulmonary Rehabilitation Program Integrating Rhythm-Guided Walking and Singing for Patients With Chronic Obstructive Pulmonary Disease: Multicenter Randomized Controlled Trial

**DOI:** 10.2196/81707

**Published:** 2026-04-27

**Authors:** Minghui Shi, Shiwei Qumu, Xiaoning Bu, Jie Zhang, Kai Liu, Jinxiang Wang, Yinan Zhang, Jieping Lei, Siyuan Wang, Wei Li, Xingyao Tang, Jisong Yan, Yaodie Peng, Chen Wang, Ting Yang, Ke Huang

**Affiliations:** 1 China-Japan Friendship Clinical Medical College, Capital Medical University, Beijing, China; 2 National Center for Respiratory Medicine, State Key Laboratory of Respiratory Health and Multimorbidity, National Clinical Research Center for Respiratory Diseases, China-Japan Friendship Hospital, Beijing, China; 3 Institute of Respiratory Medicine, Chinese Academy of Medical Sciences and Peking Union Medical College, China-Japan Friendship Hospital, Beijing, China; 4 Department of Pulmonary and Critical Care Medicine, Center of Respiratory Medicine, China-Japan Friendship Hospital, 2 Yinghua East Street, Chaoyang District, Beijing, 100029, China, 86 1084206276; 5 Department of Pulmonary and Critical Care Medicine, Beijing Tiantan Hospital, Beijing, China; 6 Department of Pulmonary and Critical Care Medicine, The Second Affiliated Hospital of Xi’an Jiaotong University, Xi' an, China; 7 Department of Rehabilitation Medicine, Qingdao Municipal Hospital, Qing Dao, China; 8 Department of Pulmonary and Critical Care Medicine, Beijing Luhe Hospital, Beijing, China; 9 Psychiatric Rehabilitation Center, Wuxi Mental Health Center, The Affiliated Mental Health Center of Jiangnan University, Wuxi, China; 10 Department of Clinical Research and Data Management, Center of Respiratory Medicine, China-Japan Friendship Hospital, Beijing, China; 11 Department of Rehabilitation Medicine, China-Japan Friendship Hospital, Beijing, China; 12 China-Japan Friendship Clinical Medical College, Peking University, Beijing, China

**Keywords:** chronic obstructive pulmonary disease, pulmonary rehabilitation, digital health, smartphone app, music

## Abstract

**Background:**

Pulmonary rehabilitation (PR) is a cornerstone for the management of chronic obstructive pulmonary disease (COPD), yet global uptake remains low due to geographic and resource barriers. Digital health technologies, specifically smartphone apps, offer a promising platform for delivering accessible home-based PR. In addition, music-assisted interventions not only offer unique physiological and psychological benefits but may also serve as an innovative approach to enhancing patient engagement and improving the effectiveness of rehabilitation in home settings.

**Objective:**

This study aimed to evaluate the effectiveness of a smartphone app–based, music-facilitated multicomponent PR program (integrating rhythm-guided walking [RW] and singing) for improving exercise capacity and other clinical outcomes in patients with COPD compared with usual care (UC).

**Methods:**

This 3-arm, parallel-group, multicenter randomized controlled trial included 70 participants in China. Participants were randomized into a multimodule training (MT) group, which included a multicomponent PR program integrating RW and singing training (n=25); a RW group, which included RW training (n=23); or a UC group (n=22). The MT and RW groups received 12-week asynchronous home-based training via a smartphone app, and all arms received structured patient education. The primary outcome was the distance achieved in the incremental shuttle walking test (ISWT) at 12 weeks. The secondary outcomes included dyspnea, quality of life, and pulmonary function.

**Results:**

The modified intention-to-treat principle was used to analyze the 70 study patients. At 12 weeks, the ISWT distance was significantly greater in the MT group than in the UC group (mean difference [MD] 56.35 m, 95% CI 6.66-106.04 m; *P*=.03; Cohen *d*=0.30). Significant improvements were observed in the MT group compared with the UC group in the modified Medical Research Council dyspnea scale (mMRC) score (MD −0.44, 95% CI −0.80 to −0.08; *P*=.02), COPD Assessment Test score (MD −3.23, 95% CI −6.18 to −0.29; *P*=.03), Hospital Anxiety and Depression Scale-anxiety subscale score (MD −2.31, 95% CI −3.99 to −0.63; *P*=.008), and inspiratory capacity (MD 15.98% predicted, 95% CI 4.76 to 27.21; *P*=.01). However, no significant differences were found between the RW and UC groups in primary or secondary outcomes. Compared with RW, MT was significantly better at decreasing the mMRC score (*P*=.03).

**Conclusions:**

The findings of this study demonstrate that our smartphone app–based music-facilitated multicomponent PR program (including tempo-guided walking and singing) caused clinically meaningful improvements in exercise capacity among patients with COPD compared to UC. Moreover, secondary outcomes, including dyspnea, quality of life, psychological status, and inspiratory capacity, showed better improvements with MT than with UC.

## Introduction

### Background

Chronic obstructive pulmonary disease (COPD) represents a major global public health challenge, which is characterized by persistent respiratory symptoms, progressive airflow limitation, and systemic manifestations, resulting in reduced physical activity, deteriorating quality of life, and psychological symptoms [1,2]. Despite advances in pharmacotherapy, many patients continue to experience substantial symptom burden and functional limitation [3]. This highlights the need for comprehensive nonpharmacological interventions, with pulmonary rehabilitation (PR) established as a cornerstone, supported by robust evidence of improved clinical outcomes [4,5]. However, traditional center-based PR remains critically underutilized globally, with only 2%‐4% of eligible patients with COPD accessing programs, and completion rates frequently below 50% [6-8]. Access is limited by multiple barriers, including a shortage of therapists, long travel distances, high treatment costs, exercise-induced dyspnea, and the monotony of conventional training [8,9].

To address these implementation gaps, digital health technologies—such as mobile health (mHealth) rehabilitation, video-based telerehabilitation, and web-based platform rehabilitation—have emerged as promising alternatives or supplements to traditional PR [10-13]. Among these, mHealth rehabilitation offers greater flexibility and reduces human resource demands compared with other modalities while simultaneously enhancing accessibility, patient engagement, and long-term adherence [14,15]. Recent reviews and randomized controlled trials (RCTs) have indicated that smartphone app–based PR programs can sustainably improve symptom scores, health-related quality of life (HRQoL), and daily physical activity levels [14-18]. However, evidence regarding objective exercise capacity remains inconsistent [14-19]. For instance, one study found that a 12-week app-based PR program significantly improved 1-minute sit-to-stand test results in patients with COPD [14], whereas another study reported no significant effect of a 12-week app-guided PR program on 6-minute walk distance in patients with chronic respiratory disease [19]. Additionally, simply digitizing the delivery of PR leaves certain patient-related barriers unaddressed. Conventional training, which primarily focuses on physical exercise, often induces fear and resistance to rehabilitation among patients with COPD owing to exertional dyspnea and fatigue. Moreover, patients often struggle to adhere because the training is perceived as monotonous and physically demanding [9].

Recent studies have identified the incorporation of music into rehabilitation training as a promising approach to address this challenge [20-25]. Receptive music can serve as a distractive auditory stimulus during aerobic exercise, facilitating auditory-motor coupling and neural entrainment to reduce perceived dyspnea and improve endurance [26,27]. Analogous to respiratory training in PR, singing or playing wind instruments promotes controlled expiratory flow, reduces dynamic hyperinflation, and strengthens respiratory muscles such as the diaphragm [28,29]. Furthermore, the inherent appeal of music itself can benefit adherence [24,25]. Nevertheless, previous research has primarily been conducted in outpatient or community care settings, with limited studies in a home-based setting [20,23,30,31]. Most studies have focused on the effects of individual music interventions, which may not adequately address the complex pathophysiological changes in COPD, including weakness of the skeletal muscles, pulmonary hyperinflation, anxiety, and depression [20,23,30-33]. The best practices for smartphone apps supporting a composite music-facilitated PR program are not yet well established.

We conducted a multicenter, 3-arm, parallel, pilot RCT to explore the preliminary efficacy and feasibility of a smartphone app–based music-facilitated multicomponent PR program with regard to clinical health outcomes in patients with COPD (COPDMELODY [Music Therapy for COPD Rehabilitation]). Prior to this study, a smartphone app (Qiyue) was developed by our research team to deliver the online music-facilitated PR program. The program integrates core PR components, including tempo-guided walking (aerobic exercise), structured singing training (respiratory training), and systematic education. Appropriate audio accompaniments and songs were carefully preselected by qualified music therapists and preinstalled in the corresponding training modules of the app. Additionally, to ensure usability and technical feasibility, the app underwent preliminary testing and feedback collection at the lead study center among a small group of patients with COPD who had prior experience with outpatient PR. The scientific rationale and detailed methodology of this music-facilitated smartphone app–based rehabilitation approach have been previously outlined in our study protocol [34].

### Objective

The objective of our study was to investigate the effectiveness of a smartphone app–based music-facilitated multicomponent PR program (incorporating rhythm-guided walking [RW] and singing) with regard to exercise capacity (primary outcome), and dyspnea, HRQoL, psychological status, and pulmonary function (secondary outcomes) in patients with COPD, compared with usual care (UC). Additionally, as an exploratory aim, we assessed the potential added benefits of combining singing training with RW versus RW alone.

## Methods

### Study Design

COPDMELODY was a multicenter, pragmatic, 3-arm, parallel-group, single-blind pilot RCT. The study protocol was registered at ClinicalTrials.gov (NCT05832814). Participants with moderate to severe stable COPD were randomized into 1 of the following 3 groups: multimodule training (MT), RW, and UC. Online training sessions were delivered over 12 weeks after randomization. Outcomes were assessed at baseline, 4 weeks, 8 weeks, and 12 weeks at hospital sites. The clinical trial protocol is presented in Multimedia Appendix 1. The CONSORT (Consolidated Standards of Reporting Trials) checklist is provided in Checklist 1.

### Participants

Participants were recruited from 5 hospitals across 3 administrative regions in China (Figure S3 and Table S11 in Multimedia Appendix 2). Participants were considered eligible if they met the following criteria: age 40-75 years, diagnosis of COPD, postbronchodilator percentage of predicted forced expiratory volume in 1 second (FEV_1_) lower than 80%, and FEV_1_ to forced vital capacity ratio lower than 0.7. Participants were required to have stable symptoms for at least 2 weeks and to be able to independently use a smartphone, as judged by their ability to perform mobile payments and use the WeChat app (Tencent). Participants were excluded if they had unstable angina or acute myocardial infarction within the past 4 weeks or had other comorbidities that would preclude participation in exercise training.

Recruitment advertisements were placed in the respiratory outpatient departments of each participating hospital, allowing interested patients to contact the research team directly. Additionally, study information leaflets were distributed to hospital medical staff, who could then refer potential candidates to the study team. Potential eligible patients who expressed interest in participation were invited to an introductory meeting—either face-to-face or via telephone—where the research team explained the study details and addressed any questions. Participation in this study was completely voluntary. Detailed descriptions of the recruitment strategies, screening process, and inclusion/exclusion criteria are provided in the study protocol [34].

### Randomization, Allocation Concealment, and Masking

Once written consent was received, a trial register form was completed, and the participant was assigned an ID number. Participants were randomly assigned to 1 of 3 groups in a 1:1:1 ratio, using a random number table generated in the SPSS statistical package (IBM Corp) by an independent statistical analyst. Randomization was conducted with permuted blocks, stratified by site. Group allocation information was stored in opaque envelopes and remained concealed until randomization occurred following the baseline assessment. Assessors and statisticians were blinded to group allocation throughout the trial; however, participants could not be blinded owing to the nature of the interventions.

### Interventions

Participants in the MT and RW groups underwent a 12-week multicomponent PR program on an individual basis at their homes through the Qiyue smartphone app. This app was specially developed for this study to deliver the PR program, including a RW module, a singing training module, and an educational module. Each module incorporated carefully preselected songs chosen by qualified music therapists to guide the corresponding training (Table S1 in Multimedia Appendix 2). The PR program for the MT group included three 30-minute RW sessions (aerobic exercise) and three 25-minute singing sessions (respiratory training) per week (total of 6 sessions per week), in addition to a standardized educational program. The PR program for the RW group only included three 30-minute RW sessions per week, in addition to the standardized educational program. The intervention was primarily asynchronous and delivered via prerecorded modules in the app, allowing patients to train at their convenience. However, the study team held a video conference with each patient during the first training session to ensure proper technique and protocol fidelity. Participants in the MT and RW groups were provided with a smartphone preinstalled with the Qiyue app, earphones, and a sports wristwatch. All devices were retrieved at the end of the 12-week intervention period.

Participants in the UC group were not provided with a smartphone (having no access to the app), but they received the same educational program as the intervention groups through WeChat messages from the research team during the study period. After the 12-week assessment, patients in the UC group were also offered an individualized PR program (a waiting list control group). Participants continued their prescribed medications (eg, inhaled drugs) throughout the study. The study team did not intervene in any physical activities the participants engaged in autonomously as part of their daily routines.

### RW Training

During RW training, patients performed RW while synchronizing their steps to music tracks with fixed tempos delivered by the Qiyue app. The intensity of walking was individualized and recalculated at baseline, week 4, and week 8, using the incremental shuttle walking test (ISWT). The calculation of the target music tempo followed a four-step process (Table 1):

1. The target walking speed was set at 75% of the maximum speed achieved during the ISWT, as recommended by the American Thoracic Society/European Respiratory Society (ATS/ERS) guidelines [4,5,35-38].
2. Individual step length was determined from the ISWT data. During the test, patients wore a camcorder to record the number of steps per shuttle. Step length was calculated by dividing the distance per shuttle (ie, 10 m) by the steps per shuttle.
3. The target step frequency was then calculated based on the target walking speed and the individual step length.
4. The music tempo was set equal to the target step frequency to guide walking.

**Table 1.** Equations for calculating individualized rhythm-guided walking tempo.

Step	Equation
1	Target walking speed (km·h^−1^) = 0.75 × peak speed in the incremental shuttle walking test (km·h^−1^)
2	Step length (m·step^−1^) = The distance per shuttle (m) ÷ the steps per shuttle
3	Target step frequency (steps·min^−1^) = Target walking speed (km·h^−1^) × 1000 ÷ step length (m·step^−1^) ÷ 60
4	Music tempo (beats·min^−1^) = Target step frequency (steps·min^−1^)

After completing the ISWT assessment, the Qiyue app automatically calculated the target tempo for each patient using the ISWT parameters and generated a personalized walking prescription (eg, three 30-minute sessions per week at a target tempo of 105 beats/min for 4 weeks). Upon returning home, participants logged into their personal account in the app to access their individualized weekly training schedule. When they opened the walking module, a series of music tracks with fixed tempos was played, and participants were instructed to walk in real time while synchronizing each step with the musical beat. Participants also wore a sports wristwatch connected to the app, which provided real-time feedback on steps, distance covered, and heart rate (Figure S1 in Multimedia Appendix 2).

### Singing Training

The singing training program consisted of 3 parts: a 5-minute video-guided breathing exercise, a 10-minute audio-guided vocal warm-up, and a 15-minute singing session. During the singing session, audio tracks of various songs were played, allowing patients to sing along in real time (Figure S2 in Multimedia Appendix 2). This structure was designed based on the latest consensus statement for Singing for Lung Health [22]. Patients’ vocals were picked up by the earphones and reported to the app when they sang.

Completed sessions, including both walking training (steps, duration, distance covered, and frequency) and singing training (vocals, duration, and frequency), were automatically recorded by the app, and aggregated data were provided to the study team to monitor and assess compliance with the intervention.

### Educational Program

The educational program covered key topics, including medication adherence, smoking cessation, maintaining physical activity, and vaccination. Participants in the MT and RW groups received 1 themed push notification per week through the Qiyue app, while those in the UC group received the same content via weekly WeChat messages sent by the study team.

### Primary Outcome

The primary outcome was the distance achieved in the ISWT, a widely recognized and frequently used measure of exercise capacity in patients with COPD. The ISWT was performed at baseline, 4 weeks, 8 weeks, and 12 weeks, according to the ATS/ERS technical standard. At each assessment, 2 tests were conducted, and the best distance was recorded to minimize the learning effect [35,39]. The primary endpoint was ISWT distance at 12 weeks after randomization. The minimal clinically important difference (MCID) for the ISWT is 35 meters [40].

### Secondary Outcomes

Several secondary outcomes were assessed. First, dyspnea symptoms were assessed using the modified Medical Research Council dyspnea scale (mMRC) at baseline and 4, 8, and 12 weeks (MCID=0.5) [41]. Second, the COPD Assessment Test (CAT) score, which indicates how symptoms impact daily life, was measured at baseline and 4, 8, and 12 weeks (MCID=1.6) [42]. Third, HRQoL was measured using the St. George’s Respiratory Questionnaire (SGRQ) and EQ-5D-5L [43]. The MCID for the SGRQ is 4 points [44]. Fourth, anxiety and depression were evaluated with the Hospital Anxiety and Depression Scale (HADS), which comprises anxiety (HADS-A) and depression (HADS-D) subscales (MCID=1.3 for the HADS-A and MCID=1.5 for the HADS-D) [45]. Fifth, lung function measurements were obtained at baseline and 12 weeks [46]. Sixth, respiratory muscle function was assessed by measuring the maximal inspiratory pressure and maximal expiratory pressure using the Gio Digital Pressure Gauge (GaleMed) [47]. Seventh, participant adherence (MT and RW groups only) was measured as the percentage of sessions completed (derived using app data) versus sessions planned [10]. Patients who completed ≥75% of the planned sessions for ≥75% of the weeks were defined as adherent users. Eighth, patient satisfaction with the app was assessed at the 12-week follow-up using a single-item global satisfaction question (“Overall, how satisfied are you with the app?”) rated on 5 levels ranging from “very dissatisfied” to “very satisfied.” Finally, adverse events, including exacerbations, exercise-related injuries, and falls, were recorded during the study period. Reliability, validity, and responsiveness of the measures have been presented in the study protocol [34].

### Sample Size

The details of the sample size calculation and statistical analysis are presented in Multimedia Appendix 2 [34]. Given the absence of previous studies exploring the effect of smartphone app–based music-facilitated multicomponent PR programs (integrating music-guided aerobic exercise and singing training), the sample size for this pilot study was calculated based on relevant studies applying similar rehabilitation interventions [48,49]. The sample size was based on the change in our primary outcome (ie, ISWT distance) from baseline to week 12. As suggested by similar studies [48,49], we tested for mean changes of 40 m, 27 m, and −15 m with combined SDs of 35 m, 38 m, and 40 m in the MT, RW, and UC groups, respectively. A sample size of 9 patients per group would yield a power of 90% at a 2-sided significance level of 5% with ANOVA. Previous RCTs evaluating outpatient training interventions reported relatively high dropout rates, ranging from 45% to 63% [12,50,51]. Therefore, assuming a conservative dropout rate of 50%, enrollment of up to 54 participants was expected (18 participants per study group). For practical purposes, considering multicenter recruitment feasibility and resource constraints, we initially planned to enroll up to 75 participants. However, due to substantial recruitment difficulties at several participating centers and limitations in time and funding (exacerbated by the COVID-19 pandemic), we were unable to enroll the originally planned number of patients in our protocol [34]. Enrollment was terminated after randomizing the 70th participant.

### Statistical Analysis

The primary analysis followed the modified intention-to-treat (mITT) principle, such that comparisons were according to the randomized group, irrespective of compliance. Categorical variables have been presented as number (percentage), and continuous variables have been presented as mean (SD). Between-group comparisons of baseline characteristics were performed using 1-way ANOVA for continuous variables and the chi-square test or Fisher exact test for categorical variables, as appropriate.

The primary outcome was evaluated by a linear mixed model (LMM) incorporating all time points, and a compound-symmetry covariance structure for the residuals was used. A single model was used for the analysis, and the primary endpoint was reported by pairwise comparisons of estimated marginal means of the 3 groups at week 12. The model included treatment group, time point of measurement, treatment by time point interaction, age, gender, BMI, and ISWT distance at baseline as fixed effects. The model included the recruitment site as a random effect. To detect differences in the proportions of participants achieving the MCID of the ISWT in the 3 groups, we also used a generalized LMM that included the same factors as the LMM described above. Evaluation of the secondary outcomes was performed analogously to the evaluation described above for the ISWT. Two sensitivity analyses were performed to evaluate the robustness of the primary analysis. One analysis excluded participants who did not complete the follow-up. A second analysis used multiple imputation to evaluate sensitivity for missing data under the assumption that data were missing at random. An iterative Markov chain Monte Carlo method was used to simulate 5 imputed values from the posterior predictive distribution of a multivariate normal model. To explore the predictors of clinical response and attrition, we performed 2 separate multivariable logistic regression models: one examining the baseline characteristics associated with achieving the MCID for ISWT distance, and another examining the factors associated with dropping out during follow-up. All statistical tests were performed at a 2-sided 5% significance level. We did not correct for multiple testing in this exploratory study. All analyses were performed in R (version 4.4.2; R Core Team).

### Ethical Considerations

This study was conducted in accordance with the Declaration of Helsinki. Ethical approval was granted by the Ethics Review Committee of the China-Japan Friendship Hospital (approval number: 2022-KY-024) and by the ethics committees of all participating centers. Written informed consent was obtained from all participants prior to enrollment. To protect participant privacy and confidentiality, all data were deidentified before analysis and storage. No financial or other compensation was provided to participants.

## Results

### Recruitment and Baseline Characteristics

**Figure 1.**
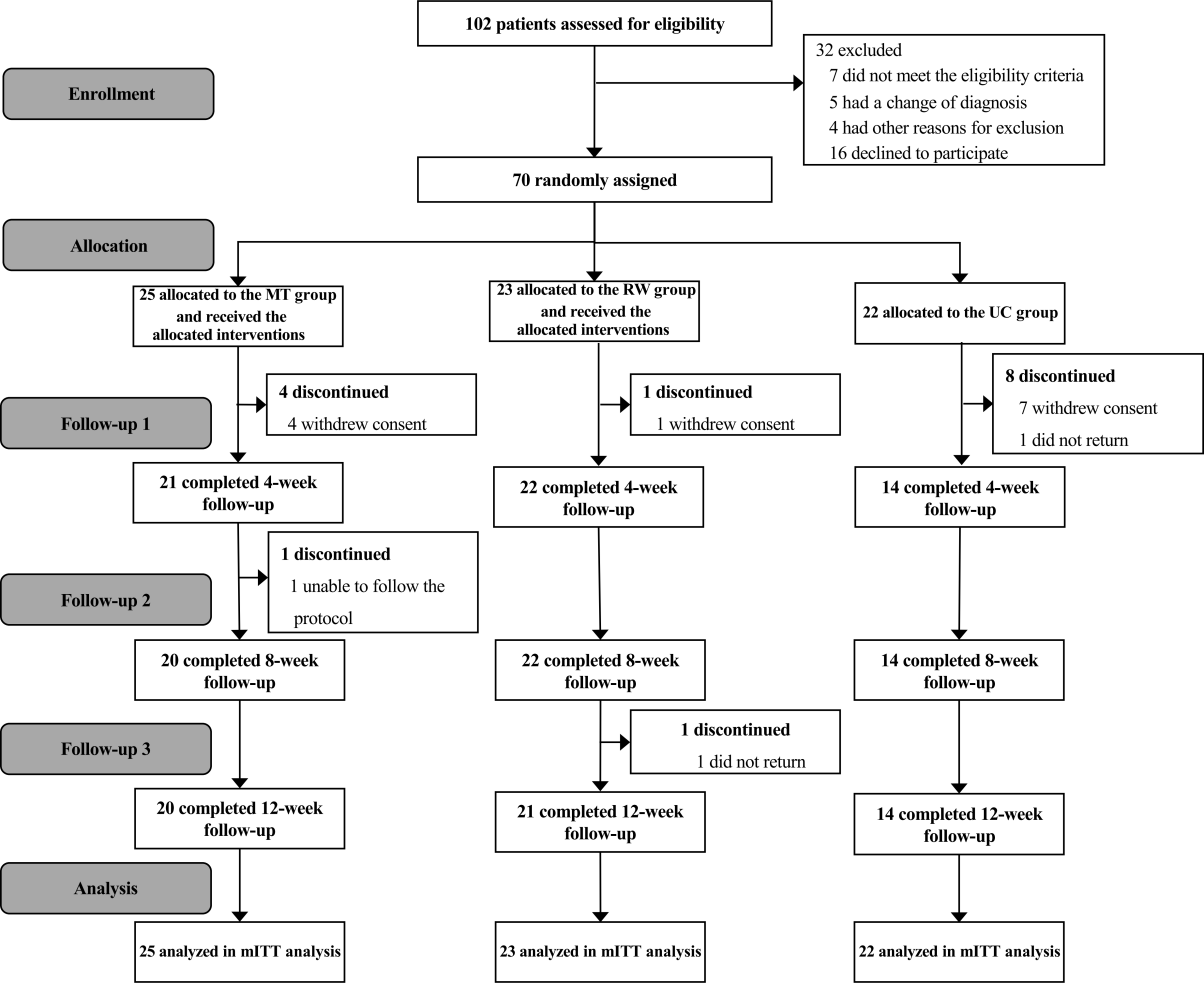
CONSORT (Consolidated Standards of Reporting Trials) flow diagram. mITT: modified intention-to-treat; MT: multimodule training; RW: rhythm-guided walking; UC: usual care.

Recruitment began in January 2023, with the first randomization occurring on February 7, 2023, and the final follow-up completed on October 23, 2023. Of the 102 patients screened, 16 were not eligible (7 failed lung function requirements; 5 had their diagnosis revised to asthma; and 4 were excluded due to unstable angina, recent exacerbation, or low digital literacy). Moreover, 16 patients did not participate in the trial for various reasons, such as “not interested” and “had no time.” Finally, 70 patients met the inclusion and exclusion criteria and were randomized into the MT, RW, or UC groups. Data from all 70 patients were included in the mITT analysis (Figure 1; Table S11 in Multimedia Appendix 2). The randomized patients with COPD had a mean age of 64.3 (SD 6.7) years and had moderate airflow obstruction, with a mean percentage of predicted FEV_1_ of 54.5% (SD 14.7%; Table 2). Of the 70 participants, 55 (79%) completed the 12-week follow-up. Attrition was higher in the UC group (8/22, 36%) than in the MT group (5/25, 20%) and RW group (2/23, 9%).

**Table 2.** Baseline characteristics of the study participants (N=70).

Variable	MT^a^ group (n=25)	RW^b^ group (n=23)	UC^c^ group (n=22)	*P* value^d^
Male, n (%)	20 (80)	16 (70)	19 (86)	.43
Age (years), mean (SD)	65.6 (5.3)	64.6 (5.3)	62.7 (9.0)	.33
BMI, mean (SD)	24.6 (3.5)	25.2 (4.4)	25.6 (2.6)	.65
Smoking history, n (%)				.79
Currently smoking	5 (20)	6 (26)	5 (23)	
Previously smoked	12 (48)	10 (44)	13 (59)	
Never smoked	8 (32)	7 (30)	4 (18)	
FEV_1_^e^ (%pred), mean (SD)	52.5 (14.2)	57.2 (12.6)	53.9 (17.5)	.53
GOLD^f^ classification, n (%)				.64
Class 2	16 (64)	16 (70)	12 (55)	
Class 3	7 (28)	7 (30)	8 (36)	
Class 4	2 (8)	0 (0)	2 (9)	
ISWT^g^ distance (m), mean (SD)	384.7 (126.6)	365.0 (123.5)	367.7 (111.6)	.83
mMRC^h^ score, mean (SD)	1.7 (0.9)	1.2 (0.4)	1.2 (0.7)	.02^i^
CAT^j^ score, mean (SD)	14.0 (6.6)	12.4 (8.1)	12.5 (5.6)	.65
Medication, n (%)				.09
LAMA^k^	7 (28)	11 (48)	2 (9)	
LABA^l^ + LAMA	5 (20)	3 (13)	4 (18)	
ICS^m^ + LABA	6 (24)	7 (30)	10 (46)	
ICS + LABA + LAMA	7 (28)	2 (9)	6 (27)	

a MT: multimodule training.

b RW: rhythm-guided walking.

c UC: usual care.

d *P* values represent overall comparisons across the 3 groups (chi-square test or Fisher exact test for categorical variables; ANOVA for continuous variables).

e FEV_1_: forced expiratory volume in 1 second.

f GOLD: Global Initiative for Chronic Obstructive Lung Disease.

g ISWT: incremental shuttle walking test.

h mMRC: modified Medical Research Council dyspnea scale.

i *P*<.05 (significant).

j CAT: COPD (chronic obstructive pulmonary disease) Assessment Test.

k LAMA: inhaled long-acting muscarinic antagonist.

l LABA: inhaled long-acting β2-agonist.

m ICS: inhaled corticosteroid.

### Exercise Capacity

**Figure 2.**
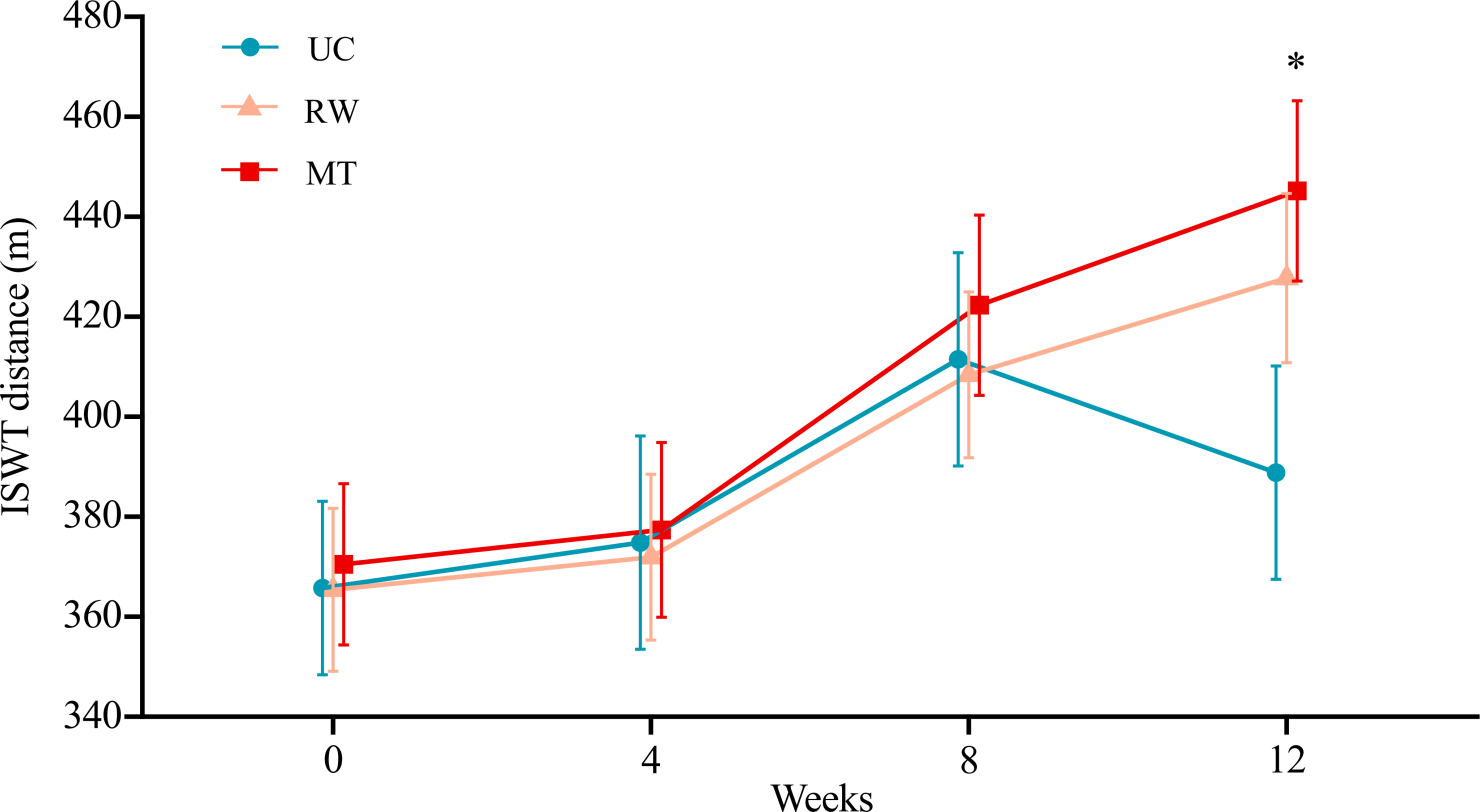
Changes in the primary outcome (incremental shuttle walking test [ISWT] distance) over 12 weeks in the multimodule training (MT), rhythm-guided walking (RW), and usual care (UC) groups. Data points show means, and error bars represent 95% CIs. *P<.05 (MT group vs UC group).

Changes in exercise capacity as measured by the ISWT over the 12-week period across the 3 groups are presented in Table 3 and Figure 2. At 12 weeks, the ISWT distance differed significantly among the 3 groups. Pairwise comparisons showed that the ISWT distance was significantly greater in the MT group than in the UC group (mean difference [MD] 56.35 m, 95% CI 6.66-106.04 m; *P*=.03; Cohen *d*=0.3), with the difference being clinically meaningful (Table 3; Table S2 in Multimedia Appendix 2). Although the RW group (the other intervention arm) showed a trend toward a greater ISWT distance compared with the UC group, the difference did not reach statistical significance (MD 38.91 m, 95% CI −10.91 to 88.74 m; *P*=.13). There was no significant difference in the ISWT distance between the MT and RW groups (*P*=.45; Table 3; Table S2 in Multimedia Appendix 2). The ISWT distance increased significantly from baseline to 12 weeks in both the MT group (*P*<.001) and RW group (*P*=.005) but not in the UC group (*P*=.39; Table 4). Additionally, the proportion of patients meeting the ISWT responder criteria was higher in the MT group (13/20, 65%) and RW group (13/21, 62%) than in the UC group (4/14, 29%; Table 5).

**Table 3.** Comparison of the ISWT^a^ distance among the MT^b^, RW^c^, and UC^d^ groups at weeks 4, 8, and 12 (modified intention-to-treat population)^e^.

Variable	Mean estimates (N=70)				MT group vs UC group		MT group vs RW group		RW group vs UC group	
	MT group (n=25)	RW group (n=23)	UC group (n=22)		Mean difference^f^ (95% CI）	*P* value^g^	Mean difference^f^ (95% CI）	*P* value^g^	Mean difference^f^ (95% CI）	*P* value^g^
ISWT distance (m)										
4 weeks	377.4	371.9	374.8		2.55 (−46.78 to 51.88)	.92	5.46 (−38.11 to 49.04)	.81	−2.29 (−52.22 to 46.39)	.91
8 weeks	422.3	408.4	411.5		10.82 (−38.87 to 60.51)	.67	13.92 (−30.34 to 58.18)	.54	−3.10 (−52.40 to 46.20)	.90
12 weeks	445.2	427.7	388.8		56.35 (6.66 to 106.04)	.03^h^	17.44 (−27.35 to 62.23)	.45	38.91 (−10.91 to 88.74)	.13

a ISWT: incremental shuttle walking test.

b MT: multimodule training.

c RW: rhythm-guided walking.

d UC: usual care.

e The linear mixed model included treatment group, time point of measurement, treatment by time point interaction, age, gender, BMI, and ISWT at baseline (fixed effects), and site (random effect).

f Differences at weeks 4, 8, and 12 between the groups are based on the differences in the marginal means of the linear mixed model.

g *P* values represent pairwise comparisons from the linear mixed model.

h *P*<.05 (significant).

**Table 4.** Changes in primary and secondary outcomes from baseline to 12 weeks in the MT^a^, RW^b^, and UC^c^ groups (N=70)^d^.

Variable		MT group (n=25)	RW group (n=23)	UC group (n=22)
Exercise capacity				
ISWT^e^ distance (m)				
Baseline, mean^f^ (SD)		370.5 (16.1)	365.4 (16.3)	365.7 (17.4)
Follow-up at 12 weeks, mean^f^ (SD)		445.2 (18.0)	427.7 (16.9)	388.8 (21.3)
Change from baseline^g^, mean (95% CI)		74.69 (31.96 to 117.42)^h^	62.35 (19.51 to 105.18)^h^	23.08 (−25.54 to 71.70)
Dyspnea				
mMRC^i^ score				
Baseline, mean^f^ (SD)		1.5 (0.2)	1.3 (0.2)	1.3 (0.2)
Follow-up at 12 weeks, mean^f^ (SD)		0.7 (0.2)	1.1 (0.2)	1.2 (0.2)
Change from baseline^g^, mean (95% CI)		−0.73 (−1.04 to −0.42)^h^	−0.21 (−0.52 to 0.10)	−0.15 (−0.50 to 0.20)
Quality of life				
CAT^j^ score				
Baseline, mean^f^ (SD)		13.0 (1.2)	12.8 (1.2)	12.8 (1.3)
Follow-up at 12 weeks, mean^f^ (SD)		7.4 (1.3)	9.9 (1.3)	10.6 (1.5)
Change from baseline^g^, mean (95% CI)		−5.65 (−8.18 to −3.12)^h^	−2.87 (−5.41 to −0.33)^h^	−2.21 (−5.10 to 0.67)
SGRQ^k^ score				
Baseline, mean^f^ (SD)		37.8 (3.1)	37.0 (3.1)	36.2 (3.3)
Follow-up at 12 weeks, mean^f^ (SD)		27.5 (3.3)	26.9 (3.2)	30.1 (3.7)
Change from baseline^g^, mean (95% CI)		−10.30 (−16.23 to −4.37)^h^	−10.07 (−16.03 to −4.11)^h^	−6.02 (−12.78 to 0.74)
EQ-5D-5L				
Baseline, mean^f^ (SD)		0.16 (0.02)	0.14 (0.02)	0.13 (0.03)
Follow-up at 12 weeks, mean^f^ (SD)		0.04 (0.03)	0.05 (0.03)	0.07 (0.03)
Change from baseline^g^, mean (95% CI)		−0.12 (−0.19 to −0.06)^h^	−0.09 (−0.15 to −0.03)^h^	−0.05 (−0.13 to 0.02)
Mental condition				
HADS-A^l^ score				
Baseline, mean^f^ (SD)		4.3 (0.7)	4.1 (0.7)	4.0 (0.7)
Follow-up at 12 weeks, mean^f^ (SD)		2.7 (0.7)	3.9 (0.7)	5.0 (0.8)
Change from baseline^g^, mean (95% CI)		−1.61 (−3.05 to −0.17)^h^	−0.21 (−1.66 to 1.24)	1.00 (−0.64 to 2.64)
HADS-D^m^ score				
Baseline, mean^f^ (SD)		4.7 (0.7)	4.6 (0.7)	4.1 (0.8)
Follow-up at 12 weeks, mean^f^ (SD)		2.8 (0.8)	3.9 (0.7)	4.3 (0.9)
Change from baseline^g^, mean (95% CI)		−1.91 (−3.50 to −0.31)^h^	−0.69 (−2.29 to 0.91)	0.18 (−1.63 to 2.00)
Respiratory muscle function				
MIP^n^ (cmH_2_O)				
Baseline, mean^f^ (SD)		79.5 (4.2)	82.5 (4.3)	79.2 (4.5)
Follow-up at 12 weeks, mean^f^ (SD)		92.2 (4.7)	82.3 (4.4)	85.5 (5.5)
Change from baseline^g^, mean (95% CI)		12.76 (2.14 to 23.38)^h^	−0.12 (−10.78 to 10.55)	6.37 (−5.73 to 18.48)
MEP^o^ (cmH_2_O)				
Baseline, mean^f^ (SD)		85.7 (5.0)	89.1 (5.0)	82.3 (5.4)
Follow-up at 12 weeks, mean^f^ (SD)		102.6 (5.5)	87.3 (5.2)	95.8 (6.5)
Change from baseline^g^, mean (95% CI)		16.87 (4.33 to 29.40)^h^	−1.80 (−14.39 to 10.79)	13.53 (−0.76 to 27.82)

a MT: multimodule training.

b RW: rhythm-guided walking.

c UC: usual care.

d The linear mixed model included treatment group, time point of measurement, treatment by time point interaction, age, gender, BMI, and ISWT at baseline (fixed effects), and site (random effect).

e ISWT: incremental shuttle walking test.

f Means at baseline and week 12 are marginal means of the linear mixed model.

g Differences between week 12 and baseline within each group are based on the differences in the marginal means of the linear mixed model.

h *P*<.05 (significant); the *P* value comparison is for the outcome at baseline vs that at week 12 within each group.

i mMRC: modified Medical Research Council dyspnea scale.

j CAT: COPD (chronic obstructive pulmonary disease) Assessment Test.

k SGRQ: St. George’s Respiratory Questionnaire.

l HADS-A: Hospital Anxiety and Depression Scale-anxiety subscale.

m HADS-D: Hospital Anxiety and Depression Scale-depression subscale.

n MIP: maximal inspiratory pressure.

o MEP: maximal expiratory pressure.

**Table 5.** Proportion of participants in the MT^a^, RW^b^, and UC^c^ groups achieving the MCID^d^ for the primary and secondary outcomes at week 12^e^.

Variable	Participants (N=55), n (%)				MT group vs UC group		MT group vs RW group		RW group vs UC group	
MT group (n=20)	RW group (n=21)	UC group (n=14)		Relative risk (95% CI)	*P* value	Relative risk (95% CI)	*P* value	Relative risk (95% CI)	*P* value
ISWT^f^	13 (65)	13 (62)	4 (29)		6.72 (1.19‐38.16)	.03	1.14 (0.26‐4.99)	.86	5.87 (1.00‐34.60)	.05
mMRC^g^	14 (70)	6 (29)	4 (29)		5.63 (1.02‐31.08)	.048	4.47 (0.95‐21.01)	.06	1.26 (0.21‐7.59)	.80
CAT^h^	14 (70)	12 (57)	10 (71)		1.99 (1.97‐2.00)	<.001	2.15 (2.12‐2.18)	<.001	0.92 (0.92‐0.93)	<.001
SGRQ^i^	14 (70)	13 (62)	7 (50)		4.34 (0.64‐29.33)	.13	1.17 (0.22‐6.28)	.85	3.71 (0.52‐26.47)	.19
HADS-A^j^	10 (50)	7 (33)	3 (21)		3.23 (0.43‐24.11)	.25	2.78 (0.46‐16.73)	.26	1.16 (0.12‐10.97)	.90
HADS-D^k^	11 (55)	8 (38)	5 (36)		3.59 (0.45‐28.90)	.23	4.72 (0.65‐34.43)	.13	0.76 (0.09‐6.10)	.80
MIP^l^	5 (25)	2 (10)	4 (29)		1.04 (0.15‐7.25)	.97	2.23 (0.22‐22.39)	.49	0.46 (0.05‐4.52)	.51

a MT: multimodule training.

b RW: rhythm-guided walking.

c UC: usual care.

d MCID: minimal clinically important difference.

e The generalized linear mixed model included treatment group, time point of measurement, treatment by time point interaction, age, gender, BMI, and baseline values (fixed effects), and site (random effect).

f ISWT: incremental shuttle walking test.

g mMRC: modified Medical Research Council dyspnea scale.

h CAT: COPD (chronic obstructive pulmonary disease) Assessment Test.

i SGRQ: St. George’s Respiratory Questionnaire.

j HADS-A: Hospital Anxiety and Depression Scale-anxiety subscale.

k HADS-D: Hospital Anxiety and Depression Scale-depression subscale.

l MIP: maximal inspiratory pressure.

### Dyspnea

**Figure 3.**
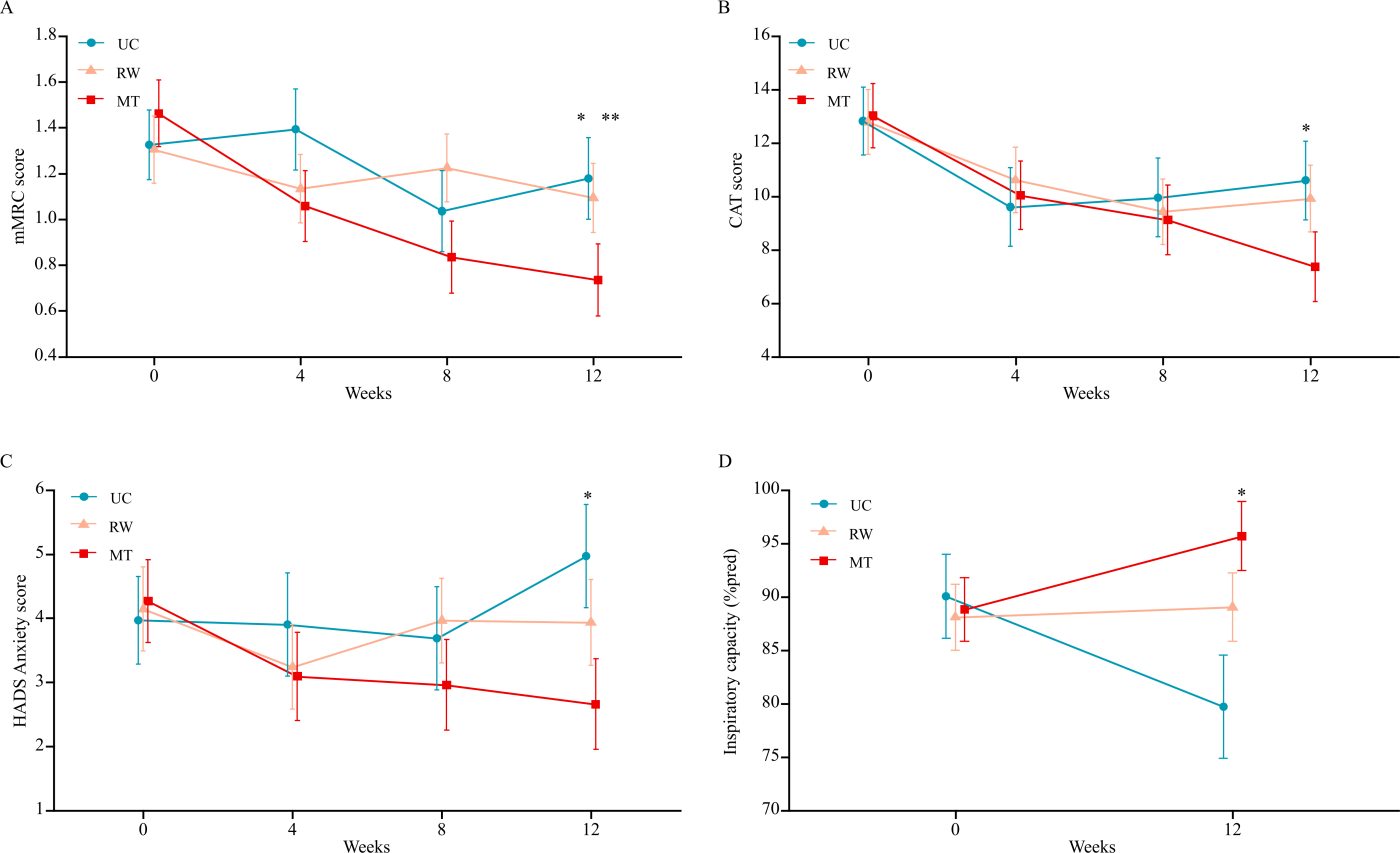
Changes in the key secondary outcomes over 12 weeks in the multimodule training (MT), rhythm-guided walking (RW), and usual care (UC) groups: (A) dyspnea symptoms (modified Medical Research Council dyspnea scale [mMRC] score), (B) quality of life (COPD Assessment Test [CAT] score), (C) anxiety symptoms (Hospital Anxiety and Depression Scale [HADS] Anxiety score), and (D) inspiratory capacity. Data points show means, and error bars represent 95% CIs. *P<.05 (MT group vs UC group), **P<.05 (MT group vs RW group).

At 12 weeks, the mMRC score was significantly lower in the MT group than in the UC group (MD −0.44, 95% CI −0.80 to −0.08; *P*=.02) and RW group (MD −0.36, 95% CI −0.69 to −0.03; *P*=.03), indicating a reduced symptom burden in the MT group. However, there was no significant difference between the RW and UC groups (*P*=.64; Table 6; Figure 3). The mMRC score decreased significantly from baseline to 12 weeks in the MT group only (*P*<.001; Table 4). Additionally, the proportion of patients achieving the MCID was higher in the MT group (14/20, 70%) than in the RW group (6/21, 29%) and UC group (4/14, 29%; Table 5). However, there was no significant difference between the RW and UC groups (*P*=.80).

**Table 6.** Comparison of the secondary outcomes among the MT^a^, RW^b^, and UC^c^ groups at week 12 (modified intention-to-treat population)^d^.

Variable	Mean estimates (N=70)			MT group vs UC group		MT group vs RW group		RW group vs UC group	
	MT group (n=25)	RW group (n=23)	UC group (n=22)	Mean difference^e^ (95% CI)	*P* value^f^	Mean difference^e^ (95% CI)	*P* value^f^	Mean difference^e^ (95% CI)	*P* value^f^
Dyspnea									
mMRC^g^	0.7	1.1	1.2	−0.44 (−0.80 to −0.08)	.02^h^	−0.36 (−0.69 to −0.03)	.03^h^	−0.09 (−0.44 to 0.27)	.64
Quality of life									
CAT^i^	7.4	9.9	10.6	−3.23 (−6.18 to −0.29)	.03^h^	−2.54 (−5.21 to 0.12)	.06	−0.69 (−3.66 to 2.28)	.65
SGRQ^j^	27.5	26.9	30.1	−2.67 (−9.59 to 4.24)	.45	0.54 (−5.70 to 6.77)	.87	−3.21 (−10.14 to 3.72)	.37
EQ-5D-5L	0.04	0.05	0.07	−0.04 (−0.11 to 0.04)	.33	−0.01 (−0.08 to 0.05)	.68	−0.02 (−0.09 to 0.05)	.55
Mental condition									
HADS-A^k^	2.7	3.9	5.0	−2.31 (−3.99 to −0.63)	.008^h^	−1.27 (−2.79 to 0.24)	.10	−1.04 (−2.72 to −0.65)	.23
HADS-D^l^	2.8	3.9	4.3	−1.44 (−3.29 to 0.42)	.13	−1.08 (−2.76 to 0.59)	.21	−0.35 (−2.21 to 1.51)	.71
Respiratory muscle function									
MIP^m^ (cmH_2_O)	92.2	82.3	85.5	6.70 (−5.68 to 19.07)	.29	9.90 (−1.28 to 21.07)	.08	−3.20 (−15.64 to 9.24)	.61
MEP^n^ (cmH_2_O)	102.6	87.3	95.8	6.73 (−7.91 to 21.38)	.37	15.24 (2.06 to 28.42)	.02^h^	−8.51 (−23.25 to 6.24)	.26
Prebronchodilator lung function (%pred)									
FEV_1_^o^	62.4	59.8	61.0	1.41 (−3.74 to 6.55)	.59	2.62 (−1.98 to 7.23)	.27	−1.22 (−6.48 to 4.04)	.65
FVC^p^	92.3	91.4	86.9	5.35 (−0.39 to 11.10)	.07	0.82 (−4.24 to 5.87)	.75	4.54 (−1.25 to 10.33)	.13
FEV_1_/FVC	51.9	50.8	48.4	3.42 (−0.81 to 7.65)	.12	1.06 (−2.74 to 4.86)	.59	2.36 (−1.94 to 6.66)	.29
PEF^q^	68.1	62.0	59.9	8.23 (−0.19 to 16.65)	.06	6.06 (−1.52 to 13.64)	.12	2.16 (−6.44 to 10.77)	.62
IC^r^	95.7	89.0	79.7	15.98 (4.76 to 27.21)	.01^h^	6.66 (−2.48 to 15.79)	.16	9.33 (−2.19 to 20.84)	.12
RV^s^	106.5	107.3	100.7	5.83 (−12.56 to 24.22)	.54	−0.80 (−17.14 to 15.53)	.92	6.63 (−12.11 to 25.38)	.49
FRC^t^	101.3	102.5	106.0	−4.71 (−15.90 to 6.47)	.41	−1.16 (−11.53 to 9.22)	.83	−3.56 (−14.84 to 7.72)	.54
TLC^u^	92.0	88.0	86.8	5.18 (−2.55 to 12.91)	.19	4.02 (−2.97 to 11.01)	.26	1.16 (−6.71 to 9.02)	.77

a MT: multimodule training.

b RW: rhythm-guided walking.

c UC: usual care.

d The linear mixed model included treatment group, time point of measurement, treatment by time point interaction, age, gender, BMI, and baseline values (fixed effects), and site (random effect).

e Differences at week 12 between the groups are based on the differences in the marginal means of the linear mixed model.

f *P* values represent pairwise comparisons from the linear mixed model.

g mMRC: modified Medical Research Council dyspnea scale.

h *P*<.05 (significant).

i CAT: COPD (chronic obstructive pulmonary disease) Assessment Test.

j SGRQ: St. George’s Respiratory Questionnaire.

k HADS-A: Hospital Anxiety and Depression Scale-anxiety subscale.

l HADS-D: Hospital Anxiety and Depression Scale-depression subscale.

m MIP: maximal inspiratory pressure.

n MEP: maximal expiratory pressure.

o FEV_1_: forced expiratory volume in 1 second.

p FVC: forced vital capacity.

q PEF: peak expiratory flow.

r IC: inspiratory capacity.

s RV: residual volume.

t FRC: functional residual capacity.

u TLC: total lung capacity.

### Assessment of HRQoL

For the SGRQ and EQ-5D-5L scores, no significant differences were observed among the 3 groups at 12 weeks (all *P*≥.05; Table 6). Nevertheless, clinically meaningful improvements from baseline to 12 weeks were observed in both the MT group (SGRQ: *P*<.001; EQ-5D-5L: *P*<.001) and RW group (SGRQ: *P*=.001; EQ-5D-5L: *P*=.004); however, changes in the UC group were not significant (SGRQ: *P*=.10; EQ-5D-5L: *P*=.14; Table 4). The proportion of participants achieving the MCID for the SGRQ did not differ significantly among the 3 groups (Table 5). For the CAT scores at 12 weeks, a significant improvement exceeding the MCID was observed in the MT group compared with the UC group (MD −3.23, 95% CI −6.18 to −0.29; *P*=.03; Table 6; Figure 3). However, there was no significant difference between the RW and UC groups (*P*=.65) or between the MT and RW groups (*P*=.06). In terms of the change in each group from baseline to 12 weeks, the CAT score improved significantly in both the MT group (*P*<.001) and RW group (*P*=.03) but not in the UC group (*P*=.19; Table 4).

### Anxiety and Depression

Regarding anxiety symptoms, pairwise comparisons revealed statistically significant differences among the 3 groups at 12 months of follow-up (Table 6; Figure 3). Specifically, the MT group demonstrated significantly greater reductions in anxiety compared with the UC group (MD −2.31, 95% CI −3.99 to −0.63; *P*=.008). There were no significant differences between the RW and UC groups (*P*=.23) or between the MT and RW groups (*P*=.10). The HADS-A score improved significantly from baseline to 12 weeks in the MT group (*P*=.03) but not in the RW group (*P*=.80) or UC group (*P*=.24; Table 4). The proportion of patients achieving the MCID for the HADS-A score was higher in both the MT group (10/20, 50%) and RW group (7/21, 33%) than in the UC group (3/14, 21%). However, these differences did not reach statistical significance (MT vs UC: *P*=.23; RW vs UC: *P*=.80; Table 5). A similar trend was observed for depression scores, though the difference across the 3 groups did not reach statistical significance (Tables5  6).

### Lung Function Measurements

Among all pulmonary function parameters assessed at 12 weeks, only inspiratory capacity (IC) showed a statistically significant difference in pairwise comparisons among the 3 groups. Specifically, IC was significantly higher in the MT group than in the UC group (MD 15.98% predicted, 95% CI 4.76%-27.21%; *P*=.007). However, no significant differences were observed between the RW and UC groups (*P*=.12) or between the MT and RW groups (*P*=.16; Table 6; Figure 3). The MT group also tended to be better at increasing the peak expiratory flow compared with the UC group at 12 weeks, though the difference was not statistically significant (*P*=.06; Table 6).

### App Adherence and Satisfaction

During the 12 weeks, except for patients who dropped out midway, all patients in the 2 intervention groups who completed the 12-week follow-up remained adherent users (MT: 20/25, 80%; RW: 21/23, 91%). The mean training session completion rate was 96.1% (SD 7.1%) in the MT group and 97.1% (SD 4.3%) in the RW group (Table S9 in Multimedia Appendix 2). Patient satisfaction with the app was very high. Except for patients who dropped out midway, all patients in the 2 intervention groups who completed the 12-week follow-up (MT: 20/25, 80%; RW: 21/23, 91%) were satisfied or very satisfied with the app (Table S10 in Multimedia Appendix 2). No participant withdrew from this study due to unresolved technical issues.

### Sensitivity Analysis

Sensitivity analyses for missing data did not show marked differences from the primary analyses (Table S6 in Multimedia Appendix 2). From the per-protocol analysis, the ISWT distance was significantly higher among participants adhering to MT than among participants receiving UC at 12 weeks (*P*=.03). There was no significant difference between the RW and UC groups (*P*=.61) or between the MT and RW groups (*P*=.46; Table S4 in Multimedia Appendix 2). The results for secondary outcomes were also similar to the findings of the mITT analysis (Table S5 in Multimedia Appendix 2).

No significant correlations were identified between baseline characteristics or other factors and achieving the MCID for the ISWT (all *P*≥.05; Table S7 in Multimedia Appendix 2). Additionally, there were no clear predictors of noncompletion among the collected characteristics (Table S8 in Multimedia Appendix 2).

## Discussion

### Principal Findings

Our study demonstrated that the use of a 12-week music-facilitated PR program (including tempo-guided walking and singing) based on mHealth technology resulted in clinically meaningful improvements in exercise capacity among patients with COPD compared to UC, with a moderate effect size [52]. Secondary outcomes also showed benefits with MT compared to UC, including improved dyspnea, quality of life, psychological status, and IC.

Our findings align with recent research on music interventions for COPD [20,22,23,30-32,53]. However, the findings are not directly comparable as previous studies involved outpatient settings or community care, and the intervention was delivered to individuals or small groups by qualified music therapists and physiotherapists [24,25]. In COPDMELODY, music-facilitated PR was home-based and provided through predesigned online programs with a smartphone app. The consistent findings suggest that home-based music interventions may be as effective as hospital- or community-based interventions targeting COPD, aided by well-designed training sessions and remote technology. This might help with the great imbalance between the large COPD population and restricted rehabilitation resources.

Following the Medical Research Council framework, the MT program represents a multicomponent complex intervention where various elements interact to produce clinical benefits [54]. The foundation of this intervention is aerobic exercise through tempo-guided walking. Within this framework, music added to exercise serves as a critical distractive auditory stimulus, shifting the patient’s focus away from an attention-demanding sensation (dyspnea) toward an external one (listening to music), which enables them to reach and maintain target exercise intensities more stably [20,21]. Singing acts as a key “active ingredient” specifically targeting respiratory mechanics. By controlling expiratory flow, lowering operating lung volumes, and doming the diaphragm to support exhalation, singing is more effective in reducing dyspnea symptoms [29]. This was supported by analyses of lung function indicating that MT was superior to UC at decreasing pulmonary hyperinflation, which is one of the main causes of dyspnea in COPD [55,56]. Furthermore, the intervention’s context was optimized through mHealth technology. The integration of smartphone apps and wearable wristwatches has been shown to reduce the complexity of delivering MT in a home setting while ensuring high protocol fidelity [14,19]. From a behavioral perspective, the inherent enjoyment and pleasure derived from the music-based program enhanced motivation and adherence [57-59], which was supported by the training data objectively measured by the app.

In contrast, RW failed to significantly improve clinical outcomes compared to UC, despite causing significant within-group improvements from baseline to 12 weeks in exercise capacity (ISWT distance) and quality of life (CAT and SGRQ scores). The findings do not align with previous studies suggesting that tempo-guided walking alone could improve exercise capacity [20,53], a discrepancy that may be attributed to the fact that the duration of walking training in those studies was substantially longer than that in our trial. Consequently, these findings suggest that the duration and frequency of walking sessions in our trial may need to be increased or supplemented with other active training modules.

Regarding the difference between MT and RW, MT demonstrated a potentially greater benefit in alleviating dyspnea compared to RW. This likely reflects the additive value of singing as a critical “active ingredient” within the complex intervention framework [22,29]. Such a multimodule approach appears to address the compound pathophysiological changes in COPD more comprehensively; however, these preliminary observations warrant further validation in future studies with adequate statistical power. We also explored the baseline predictors of achieving the MCID for the ISWT distance and those of study dropout. These exploratory analyses were conducted to identify which patients are most likely to benefit from smartphone app–based music-facilitated PR and which patients are at the greatest risk of attrition—key questions in the field. No significant predictors were identified in either analysis. The absence of clear predictors in our sample may reflect the relatively modest sample size, the homogeneity of our stable COPD cohort, and the lack of music-related personal characteristics collected. However, several previous PR studies have demonstrated that high adherence is the strongest driver of achieving the MCID for the 6-Minute Walk Test, whereas current smoking, poor lung function, and high symptom burden are associated with an increased risk of dropout [23,60]. These findings highlight the need for larger-scale and more refined future studies to better identify patient subgroups that are most likely to benefit from this kind of PR program.

### Strengths and Limitations

Our research has several advantages. COPDMELODY is the translation of research evidence for music-facilitated exercise and singing training from a hospital or community context to a home context. Most studies focus on a single kind of intervention, while an optimal rehabilitation program relies on diverse components targeting compound pathophysiological changes in COPD [33]. Following the framework for complex interventions, we innovatively leveraged the synergistic effects of combining receptive and active music interventions, enabling patients to receive the maximum benefit. Predesigned music used to guide training was provided by qualified music therapists and experienced clinicians. The training time and intensity of each patient were captured by the app and wristwatch. Besides a face-to-face course on how to perform tempo-guided walking and singing, patients had a videoconference with us during their first training session. This approach helped our trial achieve high intervention protocol fidelity [61]. To ensure the personalization and incrementality of PR, we performed the ISWT for each patient every 4 weeks [35]. Finally, this study will inform a larger trial in terms of methodology, eligibility criteria, recruitment process, sample size, randomization procedure, data collection, and data analysis process. The trial will allow for a review of the case definitions of primary outcomes, an overview of participant retention rates, and the identification of unanticipated challenges that can be addressed when preparing for a larger trial.

Our study also has some limitations. Our study was a proof-of-concept study on the impact of home-based music-facilitated PR. This trial was limited by its design and small sample size, and it lacked adequate power to demonstrate significant effects for all measured outcomes. The study made multiple comparisons between arms, which can lead to a false inference. However, as this was a pilot study, the results will not be used to make treatment decisions, but the findings can be used to generate hypotheses in subsequent, adequately powered trials. Although the final sample size exceeded the minimum target calculated in the sample size estimation, enrollment was terminated earlier than originally planned in the protocol due to practical constraints. In addition, the outcomes were evaluated over a relatively short period; thus, the intervention effect and its sustainability need to be validated in future studies. During the follow-up, the ISWT distance increased steadily among participants in the MT group but showed a fluctuating trend among participants in the UC group, whose exercise capacity might be influenced by autonomous physical activities [62]. Other clinical outcomes, including subjective and objective outcomes, supported the benefits of our PR program. Furthermore, the study population was limited to smartphone users, potentially excluding those with lower digital literacy. However, with the continuously growing number of smartphone users globally, our program has the potential to provide a supplement to or substitute (according to individual needs) for traditional center-based PR [63]. The music tracks and songs were predefined rather than based on individual choice. Although we curated “golden oldies” (1950s to 1970s) and popular hits from Chinese streaming platforms, the lack of personalization may have influenced engagement. Future research could further enhance this model by allowing participants to select music to maximize the benefits of the program.

### Conclusions

The findings of this study demonstrate that our smartphone app–based music-facilitated multicomponent PR program, involving RW and singing, has a positive effect on physiological and psychological outcomes among patients with COPD compared to UC. Specifically, exercise capacity, dyspnea, quality of life, anxiety, and IC showed significantly better improvements with MT than with UC. There is a need for further longitudinal, multicenter studies with fully powered samples to confirm sustained benefits and identify additional statistically significant events.

## Supplementary material

10.2196/81707 Multimedia Appendix 1 Clinical trial protocol.

10.2196/81707 Multimedia Appendix 2 Additional material to support the study.

10.2196/81707 Checklist 1 CONSORT checklist.

## Abbreviations

ATS/ERS: American Thoracic Society/European Respiratory Society
CAT: COPD Assessment Test
CONSORT: Consolidated Standards of Reporting Trials
COPD: chronic obstructive pulmonary disease
COPDMELODY: Music Therapy for COPD Rehabilitation
FEV_1_: forced expiratory volume in 1 second
HADS: Hospital Anxiety and Depression Scale
HADS-A: Hospital Anxiety and Depression Scale-anxiety subscale
HADS-D: Hospital Anxiety and Depression Scale-depression subscale
HRQoL: health-related quality of life
IC: inspiratory capacity
ISWT: incremental shuttle walking test
LMM: linear mixed model
MCID: minimal clinically important difference
MD: mean difference
mHealth: mobile health
mITT: modified intention-to-treat
mMRC: modified Medical Research Council dyspnea scale
MT: multimodule training
PR: pulmonary rehabilitation
RCT: randomized controlled trial
RW: rhythm-guided walking
SGRQ: St. George’s Respiratory Questionnaire
UC: usual care
