# Supplementary material for: Smartphone App–Based Music-Facilitated Pulmonary Rehabilitation Program Integrating Rhythm-Guided Walking and Singing for Patients With Chronic Obstructive Pulmonary Disease: Multicenter Randomized Controlled Trial
Source: J Med Internet Res. 2026 Apr 27;28:e81707. doi: 10.2196/81707 (PMC13119403; doi:10.2196/81707)
Supplement: Multimedia Appendix 1 [file jmir-v28-e81707-s001.doc]

**Multimedia Appendix 1.** Clinical trial protocol.

Contents

[*1 Title of the clinical trial* 3](#__RefHeading___Toc195341596)

[**1.1 Title of the clinical trial:** 3](#__RefHeading___Toc195341597)

[**1.2 Department and affiliation:** 3](#__RefHeading___Toc195341598)

[**1.3 Field of research:** 3](#__RefHeading___Toc195341599)

[*2 System for conducting research* 3](#__RefHeading___Toc195341600)

[**2.1 Study director** 4](#__RefHeading___Toc195341601)

[**2.2 In-house Investigators** 4](#__RefHeading___Toc195341602)

[**2.3 Joint Research Facilities and Investigators** 5](#__RefHeading___Toc195341603)

[*3 Introduction and objectives of the clinical trial* 6](#__RefHeading___Toc195341604)

[**3.1 Introduction:** 6](#__RefHeading___Toc195341605)

[**3.2 Objectives:** 7](#__RefHeading___Toc195341606)

[*4 Methods and duration of the study* 7](#__RefHeading___Toc195341607)

[**4.1 study design** 7](#__RefHeading___Toc195341608)

[**4.2 Study population and recruitment** 8](#__RefHeading___Toc195341609)

[**4.3** **Randomization, allocation concealment, and blinding** 9](#__RefHeading___Toc195341610)

[**4.4 Interventions** 10](#__RefHeading___Toc195341611)

[4.4.1 Multi-model MT group 10](#__RefHeading___Toc195341612)

[4.4.2 Rhythm-guided walking group 11](#__RefHeading___Toc195341613)

[4.4.3 Usual care group (Waiting-list control group) 11](#__RefHeading___Toc195341614)

[4.5 Outcome measures 12](#__RefHeading___Toc195341615)

[4.6 Data management and quality control 12](#__RefHeading___Toc195341616)

[4.7 Statistical analysis methods and sample size calculation 13](#__RefHeading___Toc195341617)

[4.7.1 Statistical analysis methods 13](#__RefHeading___Toc195341618)

[4.7.2 Sample size 14](#__RefHeading___Toc195341619)

[*5 Procedures for obtaining informed consent* 14](#__RefHeading___Toc195341620)

[*6 Handling of personal data and other information* 14](#__RefHeading___Toc195341621)

[*7 Methods of disclosing information on research* 15](#__RefHeading___Toc195341622)

[**7.1 Research registration** 15](#__RefHeading___Toc195341623)

[**7.2 Publication of research results** 15](#__RefHeading___Toc195341624)

[**7.3 Attribution of results** 15](#__RefHeading___Toc195341625)

[*8 Definition and Management of Adverse Events* 15](#__RefHeading___Toc195341626)

[**8.1 Definition of Adverse Events** 15](#__RefHeading___Toc195341627)

[**8.2 Recording and Reporting of Adverse Events** 15](#__RefHeading___Toc195341628)

[9 References 15](#__RefHeading___Toc195341629)

# **1 Title of the clinical trial**

**1.1 Title of the clinical trial:** Efficacy of a music-therapy facilitated pulmonary telerehabilitation program in patients with chronic obstructive pulmonary disease: a multicenter, randomized controlled trial (COPDMELODY trial)

**Schema (Figure 1)**

**1.2 Department and affiliation:** Department of Pulmonary and Critical Care Medicine, China-Japan Friendship Hospital, National Clinical Research Center for Respiratory Diseases, State Key Laboratory of Respiratory Health and Multimorbidity, National Center for Respiratory Medicine

**1.3 Field of research:** Respiratory medicine

# **2 System for conducting research**

□ Single-site research

■ Multicenter study (Group name: **COPDMELODY**)

■ This institution is a principal investigative institution

□ Other institution is a principal investigative institution

**Participating institutions**

1) Beijing Tiantan Hospital, Beijing, China.

2) The Second Affiliated Hospital of Xi’an Jiaotong University, Xi’an, Shanxi, China.

3) Qingdao Municipal Hospital, Qingdao, Shandong, China.

4) Beijing Luhe Hospital, Beijing, China.

5) The Affiliated Mental Health Center of Jiangnan University, Wuxi, Jiangsu, China.

**2.1 Study director (name, affiliation, official title, etc.)**

Name: **Ting Yang**, Affiliation: National Center for Respiratory Medicine, State Key Laboratory of Respiratory Health and Multimorbidity, National Clinical Research Center for Respiratory Diseases, China-Japan Friendship Hospital, Department of Pulmonary and Critical Care Medicine, Official title: Deputy Director, National Center for Respiratory Medicine

**2.2 In-house Investigators (name, affiliation, official title, etc.)**

**Co-Investigator and Research Staff**

Director (National Center for Respiratory Medicine): Chen Wang, Chief physician: Ke Huang, Chief physician: Shiwei Qumu, Deputy Chief Physiotherapist: Siyuan Wang, Clinical Fellow: Minghui Shi, Clinical Fellow: Wei Li, Clinical Fellow: Xingyao Tang, Clinical Fellow: Jisong Yan, Clinical Fellow: Yaodie Peng

**Person in Charge of Allocation**

Department of Clinical Research and Data Management: **Jieping Lei**

**Role of Study Director**

The study director is in charge of the conception, planning, and preparation of the clinical trial protocol for this clinical trial in compliance with the Declaration of Helsinki and the ethical guidelines for clinical research, also supervises all work related to the study. The study director identifies the information necessary to anticipate risks and ensure safety and effectively guarantee adequate security until the study is completed, respect the dignity and human rights of individual subjects, and take the necessary actions to protect personal information. The study director reports necessary information to the heads of the participating institutions and to the ethics committee to assess adverse events and to ensure the appropriateness and reliability of the clinical study.

**Role of Investigators (in this institution)**

Investigators conduct the study based on the plan and work content specified in the study protocol and perform the work related to the study. Investigators are responsible for protecting the life, health, privacy, and dignity of the subjects based on the expertise and clinical experience necessary to properly conduct this research. Investigators provide the subjects with sufficient explanation of the necessary matters concerning the conduct of this research, and will obtain informed consent in writing from the subjects.

**Role of person responsible for allocation**

The person responsible for allocation prepares and keeps an allocation list prior to the start of the clinical trial. The subjects are randomly grouped according to this allocation table.

**2.3 Joint Research Facilities and Investigators**

**1) Beijing Tiantan Hospital**

**Principal Investigator** (name, affiliation, official title, etc)

Name: **Xiaoning Bu**, Affiliation: Department of Pulmonary and Critical Care Medicine, Official title: Director

**Co-investigator** (name, affiliation, official title, etc)

Name: **Jing Li**, Affiliation: Department of Pulmonary and Critical Care Medicine, Official title: Clinical Fellow

**2) The Second Affiliated Hospital of Xi’an Jiaotong University**

**Principal Investigator** (name, affiliation, official title, etc)

Name: **Jie Zhang**, Affiliation: Department of Pulmonary and Critical Care Medicine, Official title: Director

**Co-investigator** (name, affiliation, official title, etc)

Name: **Hu Shan**, Affiliation: Department of Pulmonary and Critical Care Medicine, Official title: Chief physician

**3) Qingdao Municipal Hospital**

**Principal Investigator** (name, affiliation, official title, etc)

Name: **Kai Liu**, Affiliation: Department of Rehabilitation Medicine, Official title: Chief Physiotherapist

**Co-investigator** (name, affiliation, official title, etc)

Name: **Siyue Sa**, Affiliation: Department of Rehabilitation Medicine, Official title: Physiotherapist

**4) Beijing Luhe Hospital**

**Principal Investigator** (name, affiliation, official title, etc)

Name: **Jinxiang Wang**, Affiliation: Department of Pulmonary and Critical Care Medicine, Official title: Director

**Co-investigator** (name, affiliation, official title, etc)

Name: **Jie Song**, Affiliation: Department of Pulmonary and Critical Care Medicine, Official title: Chief physician

**Role of the principal investigator and participating researchers** (in Beijing Tiantan Hospital, The Second Affiliated Hospital of Xi’an Jiaotong University, Qingdao Municipal Hospital, Beijing Luhe Hospital)

The principal investigator and participating researchers are responsible for conducting the study based on the plan and work content specified in the study protocol.

**5) The Affiliated Mental Health Center of Jiangnan University**

**Principal Investigator** (name, affiliation, official title, etc)

Name: **Yinan Zhang**, Affiliation: the Affiliated Mental Health Center, Official title: music therapist

**Role of the principal investigator** (in The Affiliated Mental Health Center of Jiangnan University)

The principal investigator for is responsible for designing this clinical study, especially with regard to designing the singing training session structure and providing recorded music (for vocal exercise) and songs.

# **3 Introduction and objectives of the clinical trial**

**3.1 Introduction:** Chronic obstructive pulmonary disease (COPD) is the leading cause of morbidity and premature mortality globally, surpassing cancer, with the third-highest number of deaths in China, resulting in overwhelming pressure on healthcare systems(1). Moreover, living with COPD is often a daily struggle marked by reduced physical activity, further increasing disease progression, and creating a vicious circle (2). Psychologically, patients with COPD tend to suffer from increased stress and anxiety due to dyspnea and muscle dysfunction(3, 4).

Despite significant evidence on exercise capacity, symptoms and prognosis, the implementation of conventional pulmonary rehabilitation (PR) for COPD is insufficient, mainly due to inadequate resources, high cost, travel distance, lack of time, and low self-efficacy (5). Nonetheless, various telerehabilitation programs have been developed to overcome these barriers (6, 7). However, they play a limited role in reversing patients' low interests in exercises and rejection of rehabilitation due to fear of dyspnea and fatigue during training. Moreover, it is difficult to ensure the exact intensity of home-based exercises without professional equipment and real-time supervision (8, 9) and telerehabilitation programs with minimal equipment support are limited.

Recent studies have highlighted that music-facilitated rehabilitation not only benefits the interrelated physical and psychological consequences of patients but also improves motivation and training adherence. Music therapy (MT) targeting COPD includes passive (listening to music during aerobic exercises) and active elements (singing) (10, 11). Using rhythms to control the walking speed could offer an easy, economical way to ensure the exercise intensity at home. It can also be regarded as distractive auditory stimulus therapy, helping with dyspnea and fatigue during exertion (12). Additionally, singing promotes adaptation to breath control and training related respiratory muscles with less cost and more interest (13). Therefore, integrating tele-PR and MT may help overcome the previously mentioned barriers. However, there is a lack of relevant home-based studies as most of the existing studies were conducted in the hospital or community. Moreover there is limited evidence on the integrated effects of singing and music-guided aerobic exercises, with most studies only applying one kind of intervention (14).

Therefore, to identify a helpful and easy-to-use rehabilitation mode, our study aimed to develop a home-based, music-facilitated rehabilitation program for patients with COPD, using music tempo-guided walking exercises and breathing exercises, such as singing. Based on a thorough literature review, this is the first study to combine the two forms of MT for home-based PR and apply objective recording and supervision of training implementation using wearable sensors (a sports wristwatch that monitors step counts and walking distance) for music-facilitated tele-PR. This study protocol describes the process to investigate the efficacy and safety of the developed MT-facilitated telerehabilitation program (including rhythm-guided walking and singing).

**3.2 Objectives:** The primary aim of this study is to explore the efficacy of this home-based, MT- facilitated rehabilitation program (including rhythm-guided walking and singing). The secondary aim is to explore whether addition of singing training results in improvements compared with rhythm-guided walking alone.

# **4 Methods and duration of the study**

**4.1 study design**

This study will use a multi-center, prospective, three-arm, randomized controlled trial design. The study will include patients with COPD enrolled in a multi-module MT rehabilitation group (multi-module MT group), a rhythm-guided walking rehabilitation group (rhythm-guided walking group), and a usual care group. The study period will last for 12 weeks, and the outcomes will be measured at baseline (V0), 4 weeks (V1), 8 weeks (V2), and the end of intervention (V3). The study schedule and assessments are summarized in **Table 1.**

**Table 1.** Summary of study schedule

|  | **Enrolment** | **Baseline (V0)** | **4 weeks (V1)** | **8 weeks (V2)** | **12 weeks (V3)** |
| --- | --- | --- | --- | --- | --- |
| **Eligibility screening** |  |  |  |  |  |
| **Informed consent** | **√** |  |  |  |  |
| **Randomization** |  | **√** |  |  |  |
| **Allocation** |  | **√** |  |  |  |
| **Interventions** |  |  |  |  |  |
| **Multi-module music therapy** |  |  | | | |
| **Rhythm-guided Walking** |  |  | | | |
| **Usual care** |  |  | | | |
| **Assessments** |  |  |  |  |  |
| **Demographic characteristics** |  | **√** |  |  |  |
| **ISWT** |  | **√** | **√** | **√** | **√** |
| **MIP; MEP** |  | **√** | **√** | **√** | **√** |
| **Spirometry** |  | **√** |  |  | **√** |
| **SPPB** |  | **√** | **√** | **√** | **√** |
| **mMRC; CAT** |  | **√** | **√** | **√** | **√** |
| **SGRQ; EQ-5D** |  | **√** | **√** | **√** | **√** |
| **HADS** |  | **√** | **√** | **√** | **√** |
| **IPAQ** |  | **√** | **√** | **√** | **√** |
| **Training adherence** |  |  | **√** | **√** | **√** |
| **AEs** |  |  | **√** | **√** | **√** |

ISWT, incremental shuttle walking test; MIP, maximal inspiratory pressure; MEP, maximal expiratory pressure; SPPB, Short Physical Performance Battery; mMRC, Modified Medical Research Council; CAT, COPD assessment test; SGRQ, St. George's Respiratory Questionnaire; EQ-5D, EuroQoL-5D Questionnaire; HADS, Hospital Anxiety and Depression Scale; IPAQ, International Physical Activity Questionnaire; AE, adverse event.

**4.2 Study population and recruitment**

This multi-center study will be conducted at the China-Japan Friendship Hospital. Patients will be recruited from the China-Japan Friendship Hospital, Beijing Tiantan Hospital, Beijing Luhe Hospital, and the Second Affiliated Hospital of Xi'an Jiaotong University, Qingdao Municipal Hospital. Advertising strategies will include flyers within the hospital and professional recommendations. Potential eligible patients who are interested in participation will be invited to have either a face-to-face or telephone meeting wherein the researchers will explain the study in detail and allow time for questions. Potential patients will be asked about medical history and undergo a pulmonary function test to ensure final eligibility. The inclusion and exclusion criteria are summarized in **Table 2**. Patients who meet the eligibility criteria will be invited to sign informed consent forms and complete baseline outcome assessment.

**Table 2.** Inclusion and exclusion criteria

| **Inclusion Criteria** | **Exclusion Criteria** |
| --- | --- |
| 1. Adults aged 40−75 years. | 1. Acute myocardial infarction within 4 weeks, unstable angina, uncontrolled atrial or ventricular arrhythmia, and heart failure. |
| 2. Diagnosed with COPD of GOLD II-IV. | 2. Hypertrophic cardiomyopathy, severe valvular heart disease, severe aortic stenosis. |
| 3. Current outpatient, stable for at least 2 weeks prior to the intervention enrolment. | 3. Acute pulmonary embolism, uncontrolled asthma, respiratory failure. |
| 4. Able to use a smartphone. | 4. Comorbidity precluding exercise training (e.g., orthopedic, neurological, or cognitive conditions). |
| 5. Able to understand the purpose of the clinical trial and voluntarily participate with signed informed consent. | 5. Presence of malignant tumor. |
|  | 6. Involved in pulmonary rehabilitation programs within the past 12 months. |

**4.3 Randomization, allocation concealment, and blinding**

All patients will be randomized in a 1:1:1 ratio (adhering to block randomization within each center) into three groups: Multi-module MT, rhythm-guided walking, and usual care groups using a table of random numbers generated in the SPSS statistical package held by an independent statistical analyst. Group information will be stored in an opaque envelope and sealed thereafter. After obtaining informed consent and baseline assessment, the study coordinator will unseal the envelope to obtain the random numbers and grouping information for each participant.

Due to the nature of the intervention, neither the participants nor the exercise instructors will be blinded. However, pulmonology and rehabilitation nurses conducting outcome assessments and statistical analysts will be blinded to the assignments.

**4.4 Interventions**

All patients will continue taking routine medications, such as bronchodilators and steroid inhalers, according to their respective conditions and will maintain their regular treatment visits throughout the study period. Additionally, patients in the two intervention groups will receive two different forms of PR programs conducted by a multi-disciplinary team consisting of pulmonologists, rehabilitation physicians, pulmonology and rehabilitation nurses, music therapists, and research assistants **(Tables 3 and 4)**. The 12-week PR program is based on exercise, facilitated with music.

Home-based rehabilitation prescriptions will be conveyed by a software installed on the smartphone, containing different function modules based on the intervention group. Each patient will be provided a secure user account to log into the software.

### **4.4.1 Multi-model MT group**

Participants in the multi-model MT group will undergo two types of training: rhythm-guided walking (aerobic exercises) and singing (respiratory muscle training). Additionally, their softwares will contain two function modules: the “rhythm-guided walking” module which includes melodies with various rhythms (60−120 bpm) that can match individual walking speeds and the “singing” module which includes various songs suitable for patients to sing, which are selected and recorded by music therapists.

The participants will undertake three walking sessions of at least 30 min per week. The exercise intensity (in the form of walking speed) will be prescribed individually, according to the ISWT results. The peak walking speed (v_peak) will be assessed by the ISWT, and the targeted training speed will be set at 75%, according to the American College of Sports Medicine. A music rhythm (i.e., stride frequency) matching the target speed will be calculated using the following formula:


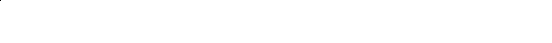
 (1)


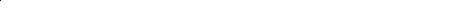
 (2)

Patients will be asked to follow this individualized music rhythm and walk at a fixed pace to maintain a constant speed. During the aerobic exercise, patients will need to open the "rhythm-guided walking" module on the software, to play the melodies with the prescribed specific tempo on a loop. Patients will need to regulate their stride frequency corresponding to the tempo to achieve adequate exercise intensity. During this, they will be asked to wear a sports wristwatch displaying their walking distance.

Additionally, the participants will undertake three singing sessions of at least 25 min per week, following the prescriptions conveyed by the software. One session includes 10 min of breathing exercises and vocal warm-up with a focus on awareness of supporting musculature during inhalation and exhalation, as well as subconscious vocal release before singing songs. It rounds up with 15 min of singing songs to improve the strength, endurance, and flexibility of the respiratory muscles. During the respiratory muscle training, patients need to open the "sing" module to follow the guiding audios and videos.

**Table 3.** Multi-module MT rehabilitation program

| **Aerobic training** |  |
| --- | --- |
| - **Rhythm-guided walking** | Exercise at an intensity of 75% of the patients' peak speed evaluated by ISWT. |
|  | Walking at a fixed pace following the music tempo matched with the targeted speed. |
| **Duration** | 30 min |
| **Respiratory muscle training** |  |
| - **Breathing exercise** - **Vocal warm-up** - **Singing songs** | Breathing control, pursed lips breathing, and abdominal breathing.  Introducing 'primal sounds' such as Hey, Ho, Ha, etc.,  Choosing appropriate songs fit for patients in terms of phrase lengths, breath points, lyrics, melodic challenge, and range. |
| **Duration** | 25 min |

MT, music therapy; ISWT, incremental shuttle walking test

### **4.4.2 Rhythm-guided walking group**

Participants in the rhythm-guided walking group will perform only music-facilitated walking exercises as previously described in the multi-module MT group.

**Table 4.** Rhythm-guided walking rehabilitation program

| **Aerobic training** |  |
| --- | --- |
| - **Rhythm-guided walking** | Exercise at an intensity of 75% of the patients' peak speed evaluated by ISWT. |
|  | Walking at a fixed pace following the music tempo matched with the targeted speed. |
| **Duration** | 30 min |

ISWT, incremental shuttle walking test

### **4.4.3 Usual care group (Waiting-list control group)**

During the study period, patients in the usual care group will not receive any rehabilitation intervention during the 12-week study period. However, they will be invited to access the music-facilitated PR program once the follow-up test is complete, thus, establishing a waiting-list control group.

**4.5 Outcome measures**

**Table 1** illustrates the items to be measured and the time window for data collection. The primary outcome is the exercise capacity measured by ISWT in all three subgroups. The ISWT will be determined according to the recommendations of the European Respiratory Society/American Thoracic Society (15). In addition, as an externally paced maximal exercise test, walking speed will be controlled by a series of pre-recorded signals. The walking speed increases progressively until the participant can no longer continue.

The secondary outcomes are:

1. Respiratory muscle function: This will be assessed by the maximal inspiratory and expiratory pressures using the Gio Digital Pressure Gauge (16).

2. Pulmonary function test: This will be assessed by spirometry performed using automated equipment as the guideline (17).

3. Lower Extremity Function: This will be assessed using the Short Physical Performance Battery (SPPB), an objective tool for measuring lower extremity physical performance status. The SPPB is based on three timed tasks: standing balance, walking speed, and chair-stand tests (18).

4. Symptoms: Chronic activity-related dyspnea will be assessed using the modified Medical Research Council dyspnea scale (mMRC). The respiratory health status will be assessed using the COPD Assessment Test (CAT).

5. Health-related quality of life (HRQoL): This will be measured using St. George's Respiratory Questionnaire (SGRQ) (19, 20) and the EuroQoL-5D Questionnaire (EQ-5D)(21). These are simple, generic HRQoL instruments widely used as patient-reported outcome measures.

6. Anxiety/depression rates: This will be evaluated in the hospital using the Hospital Anxiety and Depression Scale (HADS) which comprises seven items each for the anxiety and depression subscales. The HADS is a validated measure for assessing anxiety and depression symptoms and is recommended for patients with COPD (22, 23).

7. Physical Activity (PA) level: Daily PA will be measured using the International Physical Activity Questionnaire (IPAQ) adopted in Chinese, which presents acceptable reliability and high repeatability values (24).

8. Training adherence: Patient training adherence is defined as the percentage of the total number of completed training sessions. The supervising hospital staff will record patient adherence.

9. Safety measurements: All adverse events (AEs) that occur during the study will be recorded and evaluated for relevance to the intervention. AEs include exacerbations, exercise injuries, and falls

**4.6 Data management and quality control**

Patients will be required to practice rhythm-guided walking or singing until they master it on their first hospital visit. Telephone calls or video conferences by rehabilitation instructors will be integrated at the start of the program to ensure proper execution. After each training session, participant performance will be automatically transferred to the digital platform to facilitate the identification and verification of adherence to the prescribed exercise plan. Research assistants will verify training completion every day, record training adherence, and remind the patients to exercise.

To decrease measurement error, each participant will be assessed by a single researcher during different visits. Study data will be maintained on a password-protected platform and backed up to a secure external hard drive, with access restricted to authorized researchers and staff. Automatic plausibility controls will be set to detect any inconsistencies or inaccuracies during data entry.

**4.7 Statistical analysis methods and sample size calculation**

### **4.7.1 Statistical analysis methods**

The primary analysis will be conducted as intention-to-treat (ITT), including all patients that received randomization. Continuous baseline characteristics are given as median (range) or mean (SD) as appropriate and categorical variables as frequencies. Three comparisons will be carried out: multi-module MT versus usual care, multi-module MT versus rhythm-guided walking and rhythm-guided walking versus usual care. All analyses will be carried out using two-sided significance tests at the 0.05 significance level. Our primary analysis will compare the treatment groups at 12 weeks. The primary outcome, ISWT distance, will be analyzed using a linear mixed model, including assessments at all available time points with reference to the date of randomization. The model will include treatment group, time point of measurement as fixed effects. Other covariates treated as fixed effects were the treatment by time point interaction, age (in years), gender (male or female), BMI and ISWT at baseline. The model included recruitment site as a random effect. The model will provide treatment group differences over 12 weeks as well as estimates at individual time points. These will be presented as mean estimates with 95% confidence intervals and associated p values. For the modelling of repeated measurements, the best fitting (based on AIC and BIC information criteria), simple (not significantly different from an unstructured pattern) covariance pattern will be selected. Model assumptions will be checked and, if they are in doubt, the data will be transformed prior to analysis or alternative non-parametric analysis methods will be used. The following outcomes will be analyzed analogously to the evaluation described above for the primary outcome : mMRC scores, quality of life measures, and anxiety/depression rate, MIP/MEP and pulmonary function parameters.

Two separate sensitivity analyses will be undertaken: multiple imputation for missing data, inclusion of participants receiving the randomly assigned treatment and completing the study (i.e., a per-protocol population).

### **4.7.2 Sample size**

Considering the lack of previous studies combining music-guided walking with singing, we calculated a sample size based on studies applying similar rehabilitation interventions(25, 26). The primary outcome is the distance of ISWT and the sample size was based on change in our primary outcome from baseline to week 12. As suggested by similar studies, we assumed mean changes of multi-model MT group, rhythm-guided walking group and usual care group are 40 m, 27 m, and -15m, respectively(25, 26), and the combined standard deviations (SD) are 35 m, 38 m, and 40 m, respectively. A sample size of 9 patients per group yields a power of 90% at a 2-sided significance level of 5% with an ANOVA. Adjusting for approximately 50% dropout results in a target group size of 18 participants per study group and a total case number of 54.


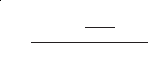


Therefore, 75 participants are anticipated to be enrolled into this study.

# **5 Procedures for obtaining informed consent**

The study will be conducted in accordance with the Declaration of Helsinki and the Ethical Guidelines for Medical Research Involving Human Subjects. The explanatory and consent documents approved by our Clinical Research Review Committee will be given to the patient, sufficient explanation will be given in writing and orally, and the patient's free and voluntary consent will be obtained in writing. When information on risks that may affect patient consent is obtained, or when changes are made to the implementation plan, etc. that may affect patient consent, the patient will be promptly informed. If there is a revision to the study and the patient must decide whether or not to participate in that study, etc., the patient's consent will be obtained again using the newly approved explanation and consent document (revised version) by the Clinical Research Review Committee of the hospital.

# **6 Handling of personal data and other information**

Persons involved in the research shall comply with the provisions of the "Ethical Guidelines for Medical Research Involving Human Subjects" as well as the "Personal Information Protection Law" and other relevant laws regarding the handling of personal information. Create data with each patient's number for the relevant medical information, and by deleting personal information create a table of correspondences between the anonymized data and the number by deleting the personal information. These data are recorded on external storage media. The anonymized data will be kept by the researcher and the corresponding table will be kept by the Principal Investigator (PI) in a locked storage room at the principal study site.

Correspondence tables will be kept by the Principal Investigator (PI) in a locked storage room at the appropriate study site. The computer on which the anonymized data and corresponding tables are stored is on a computer separate from the other computers.

# **7 Methods of disclosing information on research**

**7.1 Research registration**

This study was registered at ClinicalTrials.gov (registration number: NCT05832814).

**7.2 Publication of research results**

The results will be published in the form of a medical article. In addition, they may then also be published in the general media like the Internet.

**7.3 Attribution of results**

The results of this research shall belong to the Study director and Principal Investigators of this research, other research members and collaborators, etc., and National Center for Respiratory Medicine.

# **8 Definition and Management of Adverse Events**

**8.1 Definition of Adverse Events**

Adverse Event: An adverse event means any undesirable or unintended injury or illness or symptom thereof that occurs to a research subject, whether or not causally related to the research conducted.

Serious adverse events: Adverse events of concern in this study include pneumothorax, myalgia associated with respiratory muscle fatigue, and exacerbated dyspnea.

**8.2 Recording and Reporting of Adverse Events**

Clinical adverse events may arise during the examination of study participants. If an adverse event (including a serious adverse event) occurs, it must be thoroughly documented in the case report form, noting the time of occurrence, clinical manifestations, management steps, duration, and outcome of the event. For a serious adverse event, a serious adverse event form must be completed and immediately reported to the ethics committee.

# **9 References**

1. Wang C, Xu J, Yang L, Xu Y, Zhang X, Bai C, et al. Prevalence and risk factors of chronic obstructive pulmonary disease in China (the China Pulmonary Health [CPH] study): a national cross-sectional study. Lancet. (2018) 391**:**1706-1717. doi:10.1016/S0140-6736(18)30841-9

2. Spruit MA, Pitta F, McAuley E, ZuWallack RL, Nici L. Pulmonary Rehabilitation and Physical Activity in Patients with Chronic Obstructive Pulmonary Disease. Am J Respir Crit Care Med. (2015) 192:924-933. doi:10.1164/rccm.201505-0929CI

3. Schuler M, Wittmann M, Faller H, Schultz K. The interrelations among aspects of dyspnea and symptoms of depression in COPD patients - a network analysis. J Affect Disord. (2018) 240:33-40. doi:10.1016/j.jad.2018.07.021

4. de Voogd JN, Sanderman R, Postema K, van Sonderen E, Wempe JB. Relationship between anxiety and dyspnea on exertion in patients with chronic obstructive pulmonary disease. Anxiety Stress Coping. (2011) 24:439-449. doi:10.1080/10615806.2010.520081

5. Rochester CL, Vogiatzis I, Holland AE, Lareau SC, Marciniuk DD, Puhan MA, et al. An Official American Thoracic Society/European Respiratory Society Policy Statement: Enhancing Implementation, Use, and Delivery of Pulmonary Rehabilitation. Am J Respir Crit Care Med. (2015) 192:1373-1386. doi:10.1164/rccm.201510-1966ST

6. Holland AE, Hill CJ, Rochford P, Fiore J, Berlowitz DJ, McDonald CF. Telerehabilitation for people with chronic obstructive pulmonary disease: feasibility of a simple, real time model of supervised exercise training. J Telemed Telecare. (2013) 19:222-226. doi:10.1177/1357633x13487100

7. Stafinski T, Nagase FI, Avdagovska M, Stickland MK, Menon D. Effectiveness of home-based pulmonary rehabilitation programs for patients with chronic obstructive pulmonary disease (COPD): systematic review. BMC Health Serv Res. (2022) 22:557. doi: 10.1186/s12913-022-07779-9

8. Bamonti PM, Boyle JT, Goodwin CL, Wan ES, Silberbogen AK, Finer EB, et al. Predictors of Outpatient Pulmonary Rehabilitation Uptake, Adherence, Completion, and Treatment Response Among Male U.S. Veterans With Chronic Obstructive Pulmonary Disease. Arch Phys Med Rehabil. (2022) 103:1113-1121.e1. doi:10.1016/j.apmr.2021.10.021

9. Velez M, Lugo-Agudelo LH, Patiño Lugo DF, Glenton C, Posada AM, Mesa Franco LF, et al. Factors that influence the provision of home-based rehabilitation services for people needing rehabilitation: a qualitative evidence synthesis. Cochrane Database Syst Rev. (2023) 2:Cd014823. doi:10.1002/14651858.CD014823

10. Canga B, Azoulay R, Raskin J, Loewy J. AIR: Advances in Respiration - Music therapy in the treatment of chronic pulmonary disease. Respir Med. (2015) 109:1532-1539. doi:10.1016/j.rmed.2015.10.001

11. Panigrahi A, Sohani S, Amadi C, Joshi A. Role of music in the management of chronic obstructive pulmonary disease (COPD): a literature review. Technol Health Care. (2014) 22:53-61. doi:10.3233/THC-130773

12. Lee AL, Desveaux L, Goldstein RS, Brooks D. Distractive Auditory Stimuli in the Form of Music in Individuals With COPD: A Systematic Review. Chest. (2015) 148:417-429. doi:10.1378/chest.14-2168

13. Lewis A, Cave P, Stern M, Welch L, Taylor K, Russell J, et al. Singing for Lung Health-a systematic review of the literature and consensus statement. NPJ Prim Care Respir Med. (2016) 26:16080. doi:10.1038/npjpcrm.2016.80

14. Bausewein C, Booth S, Gysels M, Higginson IJ. Non-pharmacological interventions for breathlessness in advanced stages of malignant and non-malignant diseases. Cochrane Database Syst Rev. (2013) 11:Cd005623. doi:10.1002/14651858.CD005623.pub2

15. Holland AE, Spruit MA, Troosters T, Puhan MA, Pepin V, Saey D, et al. An official European Respiratory Society/American Thoracic Society technical standard: field walking tests in chronic respiratory disease. Eur Respir J. (2014) 44:1428-1446. doi:10.1183/09031936.00150314

16. Laveneziana P, Albuquerque A, Aliverti A, Babb T, Barreiro E, Dres M, et al. ERS statement on respiratory muscle testing at rest and during exercise. Eur Respir J. (2019) 53:1801214. doi:10.1183/13993003.01214-2018

17. Miller MR, Crapo R, Hankinson J, Brusasco V, Burgos F, Casaburi R, et al. General considerations for lung function testing. Eur Respir J. (2005) 26:153-161. doi:10.1183/09031936.05.00034505

18. de Fátima Ribeiro Silva C, Ohara DG, Matos AP, Pinto A, Pegorari MS. Short Physical Performance Battery as a Measure of Physical Performance and Mortality Predictor in Older Adults: A Comprehensive Literature Review. Int J Environ Res Public Health. (2021) 18:10612. doi:10.3390/ijerph182010612

19. Meguro M, Barley EA, Spencer S, Jones PW. Development and Validation of an Improved, COPD-Specific Version of the St. George Respiratory Questionnaire. Chest. (2007) 132:456-463. doi:10.1378/chest.06-0702

20. Xu W, Collet JP, Shapiro S, Lin Y, Yang T, Wang C, et al. Validation and clinical interpretation of the St George's Respiratory Questionnaire among COPD patients, China. Int J Tuberc Lung Dis. (2009) 13:181-189.

21. Rutten-van Mölken MP, Oostenbrink JB, Tashkin DP, Burkhart D, Monz BU. Does quality of life of COPD patients as measured by the generic EuroQol five-dimension questionnaire differentiate between COPD severity stages? Chest. (2006) 130:1117-1128. doi:10.1378/chest.130.4.1117

22. Bjelland I, Dahl AA, Haug TT, Neckelmann D. The validity of the Hospital Anxiety and Depression Scale. An updated literature review. J Psychosom Res. (2002) 52:69-77. doi:10.1016/s0022-3999(01)00296-3

23. Huang K, Huang K, Xu J, Yang L, Zhao J, Zhang X, et al. Anxiety and Depression in Patients with Chronic Obstructive Pulmonary Disease in China: Results from the China Pulmonary Health [CPH] Study. Int J Chron Obstruct Pulmon Dis. (2021) 16:3387–3396. doi:10.2147/COPD.S328617

24. Lee PH, Macfarlane DJ, Lam TH, Stewart SM. Validity of the International Physical Activity Questionnaire Short Form (IPAQ-SF): a systematic review. Int J Behav Nutr Phys Act. (2011) 8:115. doi:10.1186/1479-5868-8-115

25. Wang CH, Chou PC, Joa WC, Chen LF, Sheng TF, Ho SC, et al. Mobile-phone-based home exercise training program decreases systemic inflammation in COPD: a pilot study. BMC Pulm Med. (2014) 14:142. doi:10.1186/1471-2466-14-142

26. Leung RW, McKeough ZJ, Peters MJ, Alison JA. Short-form Sun-style t'ai chi as an exercise training modality in people with COPD. Eur Respir J. (2013) 41:1051-1057. doi:10.1183/09031936.00036912
