# Supplementary material for: Smartphone App–Based Music-Facilitated Pulmonary Rehabilitation Program Integrating Rhythm-Guided Walking and Singing for Patients With Chronic Obstructive Pulmonary Disease: Multicenter Randomized Controlled Trial
Source: J Med Internet Res. 2026 Apr 27;28:e81707. doi: 10.2196/81707 (PMC13119403; doi:10.2196/81707)
Supplement: Multimedia Appendix 2 [file jmir-v28-e81707-s002.docx]

**Multimedia Appendix 2.** Additional material to support the study.

content

[Materials for preparation 3](#_Toc223890797)

[Musical materials for exercises 3](#_Toc223890798)

[Content elements of each training 4](#_Toc223890799)

[Description of the Qiyue app 4](#_Toc223890800)

[Quality control 5](#_Toc223890801)

[Worked examples of rhythm-guided walking speed process and compliance 5](#_Toc223890802)

[Figure S1 Tempo-guided walking training of the Qiyue App 6](#_Toc223890803)

[Figure S2 Singing training of the Qiyue App 7](#_Toc223890804)

[Figure S3 The number of five participating medical centers in the COPDMELODY and their locations across China. 8](#_Toc223890805)

[Table S1 Examples of musical materials for exercises (recognizable to English readers) 9](#_Toc223890806)

[Table S2 Effectiveness of multi-module training and rhythm-guided walking on patients with COPD at 12 weeks (Modified Intention-To-Treat Population). 12](#_Toc223890807)

[Table S3 Baseline characteristics (Per-Protocol Population) 16](#_Toc223890808)

[Table S4 The ISWT distance at week 4, 8 and 12 (Per-Protocol Population) 18](#_Toc223890809)

[Table S5 The secondary outcomes at week 12 (Per-Protocol Population) 19](#_Toc223890810)

[Table S6 Mean between-group difference (95% Confidence Interval) of the MT, RW and UC groups at 4, 8, and 12 weeks for the primary outcome for the primary analysis (estimated mean difference, from Table 2 in the Manuscript) and 2 sensitivity analyses: (1) imputed (multiple imputation used to deal with missing data), (2) Per-Protocol Population. 21](#_Toc223890811)

[Table S7 Relationship between demographic, clinical, and functional variables and accomplishing minimal important difference of ISWT distance (35 meters) 22](#_Toc223890812)

[Table S8 Relationship between demographic, clinical, and functional variables and dropping out during the follow-up 23](#_Toc223890813)

[Table S9 Participants enrolled in each medical center 24](#_Toc223890814)

[Reference 24](#_Toc223890815)

# **Materials for preparation**

Patient characteristics and outcome measurements were imported into the study-specific electronic case report form (eCRF) system. Participants in the multi-module training (MT) group and the rhythm-guided walking(RW) group were supplied with a smartphone and access to the application (app) together with a sports wristwatch. The usual care (UC) group was not supplied with the smartphone (having no access to the app) and they received the same educational program as intervention groups through Wechat (Tencent, Shenzhen, China) messages from the study team. The smartphone and the wristwatch were used to implement music-facilitated trainings and collect daily training data. All study-related equipment was collected from the participants at the end of the study period.

# **Musical materials for exercises**

Musical accompaniment for the walking exercise:

Our study team selected several golden oldies of the 50s 60s & 70s and the latest hits that were popular with elderly population according to the music charts in Chinese music streaming platforms. Then we adapted the tempo of the original repertoires to 40 to 120bpm (in groups of 5bpm) using Ablteon live 10 (Ableton, Berlin, Germany) (table S1).

Songs used in singing training:

A music therapist, having prior academic professional training in voice studies, designed the singing training session structure and provided recorded music (for vocal exercise) and songs. She had participated in classes representing the ”Singing for Lung Health”/”Singing for Breathing” concept as it was delivered in the UK in 2017 prior to our study. For our study, she selected repertoires with inspiration from the best-practice approach within singing for lung patients (”Singing for Lung Health”/”Singing for Breathing”), meeting the disease-specific, pathophysiological challenges and psychosocial needs of COPD (extending outbreaths through sung phrases, improving respiratory muscle strength and co-ordination, etc.) Repertoires used were mainly from standard Chinese song books, supplemented by some Chinese popular songs from the Internet (table S1).

# **Content elements of each training**

The rhythm-guided walking training:

- Warm up exercises for joints, muscles, circulation and breathing, following videos displayed in the app.
- Tempo-guided walking exercise. A group of music with fixed tempo was played in the app, and participants were instructed to keep walking, synchronizing their steps with the tempo (figure S1).
- Cool down: Stretching of muscles; relaxation.

The singing training:

- Practicing abdominal breathing and pursed lip breathing following premade videos.
- Doing vocal exercises (vocal range and flexibility, phonation, resonation, articulation) following recorded music.
- Singing selected repertoires. Participants were suggested to sing along with the songs played in the app. Songs were mostly learned by ear/heart with dynamic lyrics displayed in the app (figure S2).

# **Description of the Qiyue app**

The *Qiyue App* is a multiplatform smartphone application developed by our study team, which is specifically used to provide the online music-facilitated trainings in this study. Each study site and the participants have their own accounts to log in the app.

The program include the following elements:

1) Walking training guided by the music tempo of premade audios.

2) Practicing methods of breathing techniques following short videos, doing vocal exercises and singing songs following premade audios.

3) Patient education based on established patient guidelines.

Selection, frequency of training, duration of training sessions, and intensity are made by health professionals in each site based on the study arm and the recommendations of current relevant guidelines. The audios of accompaniments or songs were installed into the appropriate training module of the app. After each assessment visit, the doctors could make prescriptions of different trainings based on patients’ outcome measurements through the app. Then the patients would have access to their individualized training schedules by logging in their account. The patients are presented with exercises to be completed each week on specific days but can choose whether he or she wants to complete the exercises that day or another day (three sessions a week for each type of training). The audios in each training module were played with patients following in real time during the exercises. A timer informs about the duration of the exercises. Both the walking (steps, duration and frequency) and singing training data (vocals, duration and frequency) are automatically collected by the app and uploaded to the eCRF system.

# **Quality control**

In the MT and RW, patients were required to practice tempo-guided walking or singing until they mastered it on their first hospital visit. The study team held a video conference with each patient during the first training session. The study team checked the training adherence data every day and reminded patients by messages if they missed ≥2 sessions/week. Participants in all three groups received monthly phone calls to confirm if the participant experienced any study-related issues, remind the importance of continued follow-up, express appreciation for the participant’s willingness to continue in the study. Each participant was assessed by a single researcher during different visits, decreasing measurement error. Automatic plausibility controls will be set in our eCRF system to detect any inconsistencies or inaccuracies during data entry.

# **Worked examples of rhythm-guided walking speed process and compliance**

To illustrate the individualized rhythm-guided walking process and compliance with prescribed walking speed, two representative participants are shown below.

**Case 1 (MT group, male, 64 years)**

Baseline ISWT distance: 420.5 m; Peak walking speed achieved during the ISWT: 5.5 km·h⁻¹

The number of steps per shuttle: 13

Step length = 10 ÷ 13 = 0.76 m/step

Target walking speed = 5.5 × 0.75 = 4.1 km·h⁻¹

Target step frequency = 4.1 × 1000 ÷ 0.76 ÷ 60 = 90 steps·min⁻¹

Prescribed music tempo = 90 beats·min⁻¹

Completed training sessions: 36 out of 36 planned

Average session duration: 30 minutes

Average achieved walking distance per session: 2.0 ± 0.1 km

**Case 2 (RW group, female, 52 years)**

Baseline ISWT distance: 442 m; Peak walking speed achieved during the ISWT: 6.0 km·h⁻¹

The number of steps per shuttle: 14

Step length = 10 ÷ 14 = 0.71 m/step

Target walking speed = 6.0 × 0.75 = 4.5 km·h⁻¹

Target step frequency = 4.5 × 1000 ÷ 0.71 ÷ 60 = 106 steps·min⁻¹

Prescribed music tempo = 105 beats·min⁻¹

Completed training sessions: 34 out of 36 planned

Average session duration: 30 minutes

Average achieved walking distance per session: 2.2 ± 0.1 km


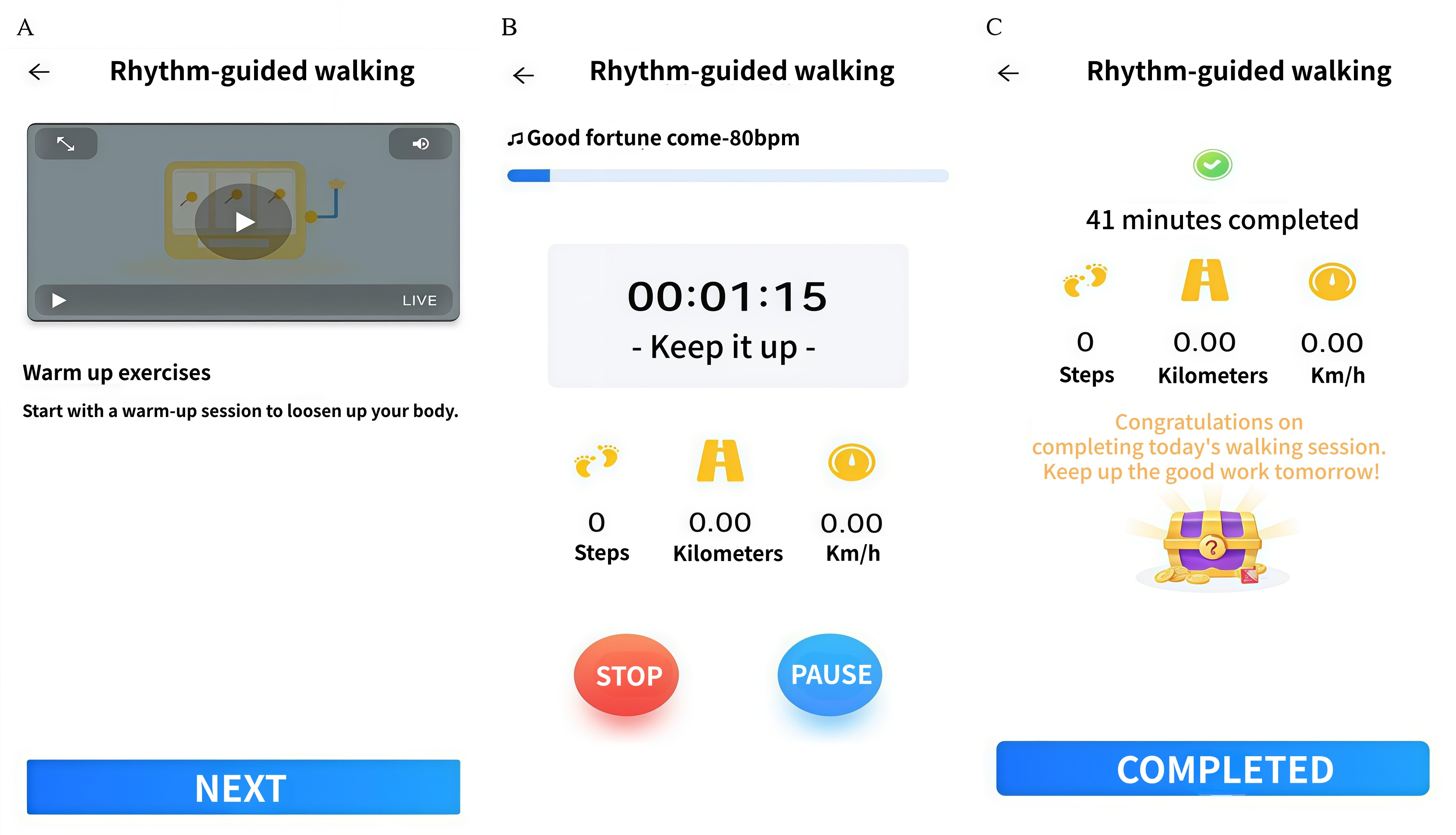


# **Figure S1 Tempo-guided walking training of the Qiyue App**


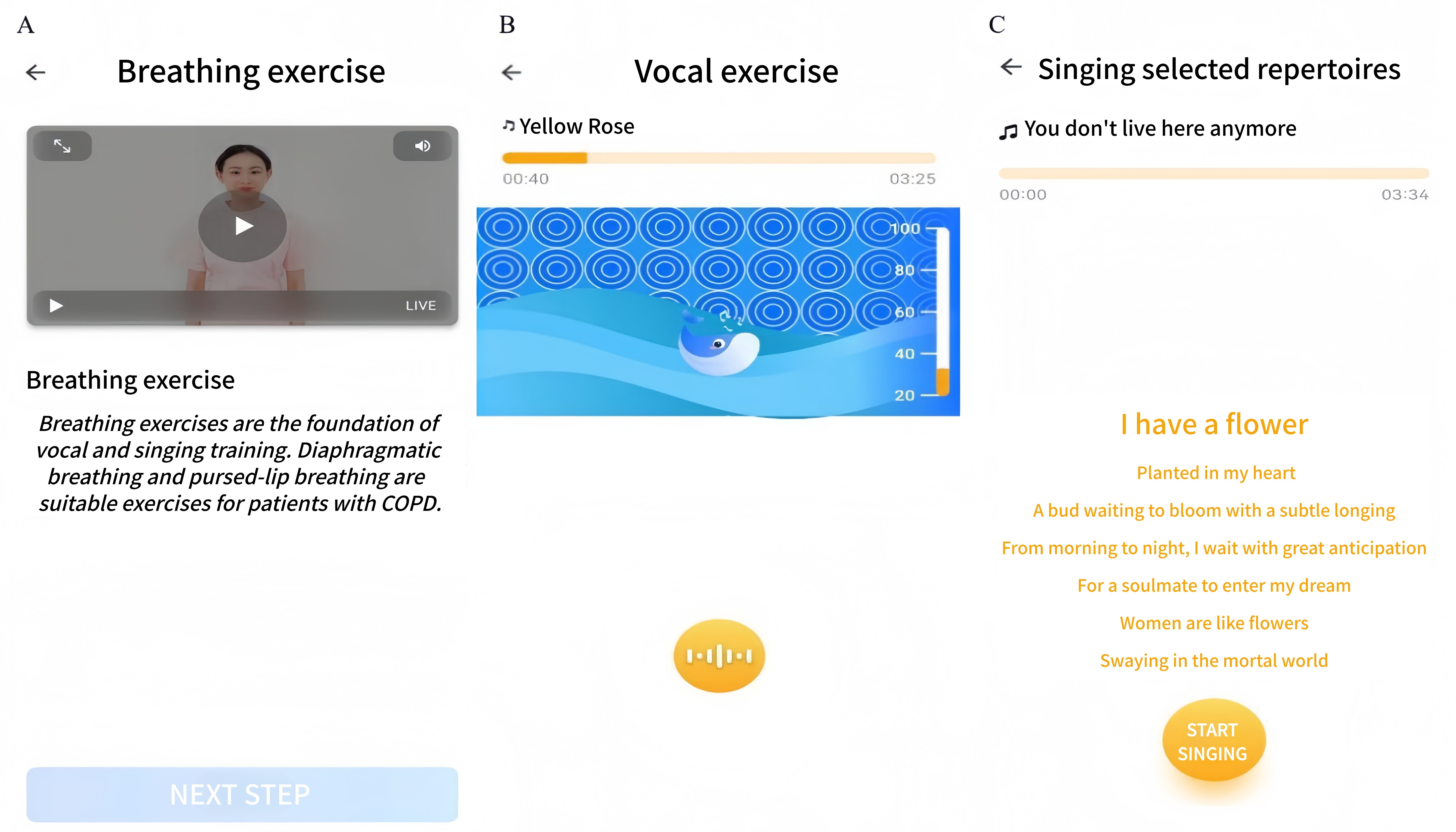


# **Figure S2 Singing training of the Qiyue App**

# **Figure S3 The locations of five participating medical centers in the COPDMELODY across China.**

Beijing enrolled three medical centers. Shandong and Shanxi enrolled one medical center each.

# **Table S1 Examples of musical materials for exercises (recognizable to English readers)**

| Musical accompaniment for the walking exercise | | Songs used in singing training |
| --- | --- | --- |
| 80bpm | Red sun (in Chinese: “红日”) | Kangding Love Song (in Chinese: “康定情歌”) |
|  | Good fortune come (in Chinese: “好运来”) | My Motherland and Me (in Chinese: “我和我的祖国”) |
| 85bpm | A Life of Fighting Is but a Dream (in Chinese: “刀剑如梦”) | Jasmine Flower (in Chinese: “茉莉花”) |
|  | The Fiery-Red Sarilang (in Chinese: “火红的萨日朗”) | You don't live here anymore (in Chinese: “女人花”) |
| 90bpm | Congratulations on getting rich (in Chinese: “恭喜发财”) |  |
|  | Intoxicated Butterfly (in Chinese: “酒醉的蝴蝶”) |  |
| 95bpm | Fine Wine with Coffee (in Chinese: “美酒加咖啡”) |  |
|  | Gimme Gimme Gimme (in Chinese: “恼人的秋风”) |  |
| 100bpm | Club Broken Heart (in Chinese: “失恋阵线联盟”) |  |
|  | Past days are what I can recall (in Chinese: “往事只能回味”) |  |
| 105bpm | Intoxicated Butterfly (in Chinese: “酒醉的蝴蝶”) |  |
|  | Little Apple (in Chinese: “小苹果”) |  |
| 110bpm | Congratulations on getting rich (in Chinese: “恭喜发财”) |  |
|  | Little Apple (in Chinese: “小苹果”) |  |
| 115bpm | A Life of Fighting Is but a Dream (in Chinese: “刀剑如梦”) |  |
|  | Gimme Gimme Gimme (in Chinese: “恼人的秋风”) |  |
| 120bpm | Congratulations on getting rich (in Chinese: “恭喜发财”) |  |
|  | Intoxicated Butterfly (in Chinese: “酒醉的蝴蝶”) |  |

# **Table S2 Effectiveness of multi-module training and rhythm-guided walking on patients with COPD at 12 weeks (Modified Intention-To-Treat Population).**

|  | MT vs UC | | | RW vs UC | | |
| --- | --- | --- | --- | --- | --- | --- |
|  | Mean difference  （95%CI） | Cohen’s d | *P* | Mean difference  （95%CI） | Cohen’s d | *P* |
| Exercise capacity |  |  |  |  |  |  |
| ISWT, m | 56.35 (6.66 to 106.04) | 0.30 | 0.03 | 38.91 (-10.91 to 88.74) | 0.21 | 0.13 |
| Dyspnea |  |  |  |  |  |  |
| mMRC | -0.44 (-0.80--0.08) | 0.33 | 0.02 | -0.09 (-0.44-0.27) | 0.06 | 0.64 |
| Quality of life |  |  |  |  |  |  |
| CAT | -3.23 (-6.18--0.29) | 0.29 | 0.03 | -0.69 (-3.66-2.28) | 0.06 | 0.65 |
| SGRQ | -2.67 (-9.59-4.24) | 0.10 | 0.45 | -3.21 (-10.14-3.72) | 0.12 | 0.37 |
| EQ-5D-5L | -0.04 (-0.11-0.04) | 0.13 | 0.33 | -0.02 (-0.09-0.05) | 0.08 | 0.55 |
| Mental condition |  |  |  |  |  |  |
| HADS-A | -2.31 (-3.99--0.63) | 0.37 | 0.008 | -1.04 (-2.72--0.65) | 0.16 | 0.23 |
| HADS-D | -1.44 (-3.29 -0.42) | 0.21 | 0.13 | -0.35 (-2.21-1.51) | 0.05 | 0.71 |
| Respiratory muscle function |  |  |  |  |  |  |
| MIP, cmH_2_O | 6.70 (-5.68-19.07) | 0.14 | 0.29 | -3.20 (-15.64-9.24) | 0.07 | 0.61 |
| MEP, cmH_2_O | 6.73 (-7.91-21.38) | 0.12 | 0.37 | -8.51(-23.25-6.24) | 0.15 | 0.26 |
| Pre-bronchodilator lung function, %pred |  |  |  |  |  |  |
| FEV_1_, | 1.41 (-3.74-6.55) | 0.10 | 0.59 | -1.22 (-6.48-4.04) | 0.09 | 0.65 |
| FVC | 5.35 (-0.39-11.10) | 0.35 | 0.07 | 4.54 (-1.25-10.33) | 0.29 | 0.13 |
| FEV_1_/FVC | 3.42 (-0.81-7.65) | 0.30 | 0.12 | 2.36 (-1.94-6.66) | 0.20 | 0.29 |
| PEF | 8.23 (-0.19-16.65) | 0.36 | 0.06 | 2.16 (-6.44-10.77) | 0.09 | 0.62 |
| IC | 15.98 (4.76-27.21) | 0.61 | 0.01 | 9.33 (-2.19-20.84) | 0.35 | 0.12 |
| RV | 5.83 (-12.56-24.22) | 0.13 | 0.54 | 6.63 (-12.11-25.38) | 0.15 | 0.49 |
| FRC | -4.71 (-15.90-6.47) | 0.21 | 0.41 | -3.56 (-14.84-7.72) | 0.15 | 0.54 |
| TLC | 5.18 (-2.55-12.91) | 0.30 | 0.19 | 1.16 (-6.71-9.02) | 0.06 | 0.77 |

Linear mixed model included treatment group, time point of measurement, treatment by time point interaction, age, gender, body mass index, baseline values (fixed effects), and site (random effect). Estimated mean differences and effect sizes (Cohen’s d) for primary and secondary outcomes at 12 weeks were calculated from the linear mixed model. MT = multi-module training; RW = rhythm-guided walking; UC = usual care. mMRC = modified Medical Research Council dyspnea scale; CAT = COPD Assessment Test; SGRQ = St. George’s Respiratory Questionnaire; EQ-5D-5L = European Quality of Life Five Dimension Five Level Scale Questionnaire; HADS-A = Hospital Anxiety and Depression Scale-Anxiety score; HADS-D = Hospital Anxiety and Depression Scale-Depression core; MIP = maximal inspiratory pressure; MEP = maximal expiratory pressure; FEV_1_= forced expiratory volume in 1 second; FVC = forced vital capacity; PEF = peak expiratory flow; IC = inspiratory capacity; RV= residual volume; FRC = functional residual capacity; TLC = total lung capacity.

# **Table S3 Baseline characteristics (Per-Protocol Population)**

|  | MT  (n=20) | RW  (n=21) | UC  (n=14) | *P* |
| --- | --- | --- | --- | --- |
| Male, % | 18 (90.0) | 14 (66.7) | 14 (100.0) | 0.03 |
| Age, years | 65.1 ± 5.7 | 65.0 ± 5.3 | 63.5 ± 8.7 | 0.74 |
| BMI, kg/m^2^ | 24.8 ± 3.5 | 25.3 ± 4.6 | 26.3 ± 2.6 | 0.53 |
| Smoking history |  |  |  | 0.55 |
| Currently smoke | 3 (15.0) | 6 (28.6) | 4 (28.6) |  |
| Previously smoked | 11 (55.0) | 8 (38.1) | 8 (57.1) |  |
| Never smoke | 6 (30.0) | 7 (33.3) | 2 (14.3) |  |
| FEV_1_, %pred | 52.5 ± 14.2 | 57.2 ± 12.6 | 53.9 ± 17.5 | 0.53 |
| GOLD classification |  |  |  | 0.25 |
| Class 2 | 13 (65.0) | 15 (71.4) | 7 (50.0) |  |
| Class 3 | 5 (25.0) | 6 (28.6) | 7 (50.0) |  |
| Class 4 | 2 (10.0) | 0 (0.0) | 0 (0.0) |  |
| ISWT distance, m | 357.2 ± 105.8 | 349.1 ± 115.1 | 372.5 ± 99.5 | 0.82 |
| mMRC | 1.7 ± 0.9 | 1.2 ± 0.4 | 1.2 ± 0.7 | 0.05 |
| CAT | 14.1 ± 6.2 | 11.3 ± 7.5 | 13.2 ± 6.3 | 0.44 |
| Medication |  |  |  | 0.31 |
| LAMA | 4 (20.0) | 9 (42.9) | 2 (14.3) |  |
| LABA+LABA | 4 (20.0) | 3 (14.3) | 4 (28.6) |  |
| ICS+LABA | 5 (25.0) | 7 (33.3) | 4 (28.6) |  |
| ICS+LABA+LAMA | 7 (35.0) | 2 (9.5) | 4 (28.6) |  |

Data are presented as No. (%) or mean ± SD. Pre was defined as pre-bronchodilator. MT = multi-module training; RW = rhythm-guided walking; UC = usual care. BMI = body mass index; FEV_1_ = forced expiratory volume in 1s; GOLD = Global Initiative for Chronic Obstructive Lung Disease; ISWT = incremental shuttle walking test; mMRC = modified Medical Research Council dyspnea scale; CAT = COPD Assessment Test; LAMA = inhaled long-acting muscarinic antagonists ; LABA = inhaled long-acting β2-agonists; ICS = inhaled corticosteroids.

# **Table S4 The ISWT distance at week 4, 8 and 12 (Per-Protocol Population)**

|  | Mean Estimates | | | | | | | MT vs UC | | | | MT vs RW | | | | RW vs UC | | | |
| --- | --- | --- | --- | --- | --- | --- | --- | --- | --- | --- | --- | --- | --- | --- | --- | --- | --- | --- | --- |
|  | MT  (n=20) | | | RW  (n=21) | | UC  (n=14) | | Mean difference  （95%CI） | | | *P* | Mean difference  （95% CI） | | | *P* | Mean difference  （95% CI） | | | *P* |
| ISWT distance, m | |  |  | |  | |  | |  |  | | |  |  | | |  |  |  |
| 4 weeks | 373.2 | | | 372.8 | | 367.4 | | 5.74 (-44.54-56.02) | | | 0.82 | 0.39 (-45.00-45.78) | | | 0.99 | 5.35 (-45.26-55.95) | | | 0.84 |
| 8 weeks | 415.8 | | | 408.4 | | 404.1 | | 11.76 (-38.52-62.04) | | | 0.65 | 7.48 (-37.91-52.87) | | | 0.75 | 4.28 (-46.32-54.89) | | | 0.87 |
| 12 weeks | 438.7 | | | 421.5 | | 381.4 | | 57.29 (7.01-107.57) | | | 0.03 | 17.24 (-28.15-62.64) | | | 0.46 | 40.05 (-10.56- 90.65) | | | 0.12 |

Linear mixed model included treatment group, time point of measurement, treatment by time point interaction, age, gender, body mass index, ISWT at baseline (fixed effects), and site (random effect). The differences at week 4, 8 and 12 between the groups are based on the difference of marginal means of the linear mixed model. MT = multi-module training; RW = rhythm-guided walking; UC = usual care. ISWT = incremental shuttle walking test.

# **Table S5 The secondary outcomes at week 12 (Per-Protocol Population)**

|  | Mean Estimates | | | MT vs UC | | MT vs RW | | | | RW vs UC | |
| --- | --- | --- | --- | --- | --- | --- | --- | --- | --- | --- | --- |
|  | MT  (n=20) | RW  (n=21) | UC  (n=14) | Mean difference  （95%CI） | *P* | | Mean difference  （95%CI） | *P* | | Mean difference  （95%CI） | *P* |
| Dyspnea |  |  |  |  |  | |  |  | |  |  |
| mMRC | 0.8 | 1.1 | 1.2 | -0.46 (-0.82--0.09) | 0.02 | | -0.34 (-0.67--0.01) | 0.046 | | -0.12 (-0.48-0.25) | 0.53 |
| Quality of life |  |  |  |  |  | |  |  | |  |  |
| CAT | 7.3 | 9.8 | 10.6 | -3.27 (-6.26--0.27) | 0.03 | | -2.53 (-5.24-0.18) | 0.07 | | -0.74 (-3.77-2.30) | 0.64 |
| SGRQ | 27.1 | 26.5 | 29.9 | -2.77 (-9.72-4.17) | 0.44 | | 0.66 (-5.62-6.94) | 0.84 | | -3.43 (-10.42-3.56) | 0.34 |
| EQ-5D-5L | 0.04 | 0.05 | 0.08 | -0.04 (-0.11-0.04) | 0.34 | | -0.01 (-0.08-0.05) | 0.72 | | -0.02 (-0.10-0.05) | 0.53 |
| Mental condition |  |  |  |  |  | |  |  | |  |  |
| HADS-A | 2.6 | 3.8 | 4.9 | -2.33 (-4.00--0.67) | 0.007 | | -1.25 (-2.75-0.26) | 0.11 | | -1.10 (-2.76--0.59) | 0.21 |
| HADS-D | 2.8 | 3.9 | 4.3 | -1.47 (-3.35-0.41) | 0.13 | | -1.06 (-2.76-0.64) | 0.22 | | -0.41 (-2.31-1.48) | 0.67 |
| Respiratory muscle function |  |  |  |  |  | |  |  | |  |  |
| MIP, cmH_2_O | 92.8 | 82.6 | 86.3 | 6.51 (-6.18-19.20) | 0.32 | | 10.19 (-1.29-21.67) | 0.08 | | -3.68 (-16.48-9.12) | 0.57 |
| MEP, cmH_2_O | 102.4 | 87.3 | 95.5 | 6.89 (-8.17-21.96) | 0.37 | | 15.08 (1.50-28.66) | 0.03 | | -8.19 (-23.42-7.05) | 0.29 |
| Pre-bronchodilator lung function, %pred |  |  |  |  |  | |  |  | |  |  |
| FEV_1_, | 64.0 | 60.5 | 62.2 | 1.82 (-3.67-7.31) | 0.52 | | 3.52 (-1.45-8.49) | 0.17 | -1.70 (-7.32-3.93) | | 0.56 |
| FVC | 85.8 | 84.8 | 85.7 | 6.02 (-0.03-12.07) | 0.05 | | 2.01 (-3.384-7.40) | 0.47 | 4.01 (-2.09-10.11) | | 0.20 |
| FEV_1_/FVC | 52.4 | 51.5 | 49.1 | 3.34 (-1.21-7.90) | 0.15 | | 0.93 (-3.23-5.09) | 0.66 | 2.41 (-2.26-7.08) | | 0.31 |
| PEF | 69.5 | 63.2 | 60.6 | 8.96 (0.11-17.80) | 0.05 | | 6.34 (-1.76-14.43) | 0.13 | 2.62 (-6.48-11.72) | | 0.57 |
| IC | 91.1 | 86.5 | 76.1 | 14.93 (3.96-25.91) | 0.009 | | 4.56 (-4.63-13.76) | 0.33 | 10.37 (-0.72-21.46) | | 0.07 |
| RV | 117.7 | 109.4 | 107.5 | 10.22 (-7.43-27.88) | 0.26 | | 8.35(-7.63-24.33) | 0.31 | 1.88 (-16.32-20.07) | | 0.84 |
| FRC | 100.0 | 101.0 | 104.6 | -4.64 (-16.62-7.34) | 0.45 | | -1.04(-12.41-10.33) | 0.86 | -3.60 (-15.95-8.76) | | 0.57 |
| TLC | 92.3 | 88.0 | 87.2 | 5.13 (-3.04-13.31) | 0.22 | | 4.29 (-3.14-11.72) | 0.26 | | 0.84 (-7.59-9.28) | 0.85 |

Linear mixed model included treatment group, time point of measurement, treatment by time point interaction, age, gender, body mass index, baseline values (fixed effects), and site (random effect). The differences at week 12 between the groups are based on the difference of marginal means of the linear mixed model. MT = multi-module training; RW = rhythm-guided walking; UC = usual care. mMRC = modified Medical Research Council dyspnea scale; CAT = COPD Assessment Test; SGRQ = St. George’s Respiratory Questionnaire; EQ-5D-5L = European Quality of Life Five Dimension Five Level Scale Questionnaire; HADS-A = Hospital Anxiety and Depression Scale-Anxiety score; HADS-D = Hospital Anxiety and Depression Scale-Depression core; MIP = maximal inspiratory pressure; MEP = maximal expiratory pressure; FEV_1_= forced expiratory volume in 1 second; FVC = forced vital capacity; PEF = peak expiratory flow; IC = inspiratory capacity; RV= residual volume; FRC = functional residual capacity; TLC = total lung capacity.

# **Table S6 Mean between-group difference (95% Confidence Interval) of the MT, RW and UC groups at 4, 8, and 12 weeks for the primary outcome for the primary analysis (estimated mean difference, from Table 2 in the Manuscript) and 2 sensitivity analyses: (1) imputed (multiple imputation used to deal with missing data), (2) Per-Protocol Population.**

|  | Sensitivity analysis | Mean between-group difference | | |
| --- | --- | --- | --- | --- |
|  |  | MT vs UC | MT vs RW | RW vs UC |
| ISWT distance, m |  |  |  |  |
| 4 weeks | difference | 2.55 (-46.78-51.88) | 5.46 (-38.11-49.04) | -2.29 (-52.22-46.39) |
|  | imputed | -5.07 (-48.48-38.35) | 5.59 (-37.10-48.29) | -10.66 (-54.90-33.58) |
|  | per-protocol | 5.74 (-44.54-56.02) | 0.39 (-45.00-45.78) | 5.35 (-45.26-55.95) |
| 8 weeks | difference | 10.82 (-38.87-60.51) | 13.92 (-30.34-58.18) | -3.10 (-52.40-46.20) |
|  | imputed | 10.45 (-32.96-53.86) | 6.33 (-36.36-49.03) | 4.12 (-40.12-48.36) |
|  | per-protocol | 11.76 (-38.52-62.04) | 7.48 (-37.91-52.87) | 4.28 (-46.32-54.89) |
| 12 weeks | difference | 56.35 (6.66-106.04) | 17.44 (-27.35-62.23) | 38.91 (-10.91-88.74) |
|  | imputed | 49.09 (5.68-92.50) | 19.32 (-23.38-62.01) | 29.77 (-14.47-74.01) |
|  | per-protocol | 57.29 (7.01-107.57) | 17.24 (-28.15-62.64) | 40.05 (-10.56-90.65) |

MT = multi-module training; RW = rhythm-guided walking; UC = usual care. ISWT = incremental shuttle walking test.

# **Table S7 Relationship between demographic, clinical, and functional variables and accomplishing minimal important difference of ISWT distance (35 meters)**

**Multivariable logistic regression analysis of baseline factors associated with achieving the MCID for ISWT distance (35 meters) at 12 weeks in COPD patients.**

|  | OR | 95%CI | *P* |
| --- | --- | --- | --- |
| Gender |  |  |  |
| Male | 1.00 | reference |  |
| Female | 1.08 | 0.22-5.33 | 0.92 |
| Age, years |  |  |  |
| <65 | 1.00 | reference |  |
| ≥65 | 1.27 | 0.36-4.48 | 0.71 |
| BMI, kg/m^2^ |  |  |  |
| <24 | 1.00 | reference |  |
| ≥24 | 3.54 | 0.95-13.20 | 0.06 |
| mMRC |  |  |  |
| ≤1 | 1.00 | reference |  |
| >1 | 0.50 | 0.13-1.87 | 0.303 |
| FEV_1_, %pred |  |  |  |
| >50 | 1.00 | reference |  |
| ≤50 | 0.41 | 0.11-1.56 | 0.19 |
| ISWT distance at baseline, m |  |  |  |
| >350 | 1.00 | reference |  |
| ≤350 | 0.72 | 0.20-2.54 | 0.61 |

Odds ratios (ORs) were computed using multivariable logistic regression. ORs > 1 indicate an increased probability of reaching the MCID of the ISWT. BMI = body mass index; FEV_1_ = forced expiratory volume in 1s; MCID = minimal clinically important difference; mMRC = modified Medical Research Council dyspnea scale; ISWT = incremental shuttle walking test.

# **Table S8 Relationship between demographic, clinical, and functional variables and dropping out during the follow-up**

**Multivariable logistic regression analysis of baseline factors associated with dropping out during the follow-up in COPD patients.**

|  | OR | 95%CI | *P* |
| --- | --- | --- | --- |
| Gender |  |  |  |
| Male | 1.00 | reference |  |
| Female | 0.29 | 0.08-1.03 | 0.056 |
| Age, years |  |  |  |
| <65 | 1.00 | reference |  |
| ≥65 | 0.93 | 0.29-2.97 | 0.90 |
| BMI, kg/m^2^ |  |  |  |
| <24 | 1.00 | reference |  |
| ≥24 | 2.23 | 0.67-7.38 | 0.19 |
| mMRC |  |  |  |
| >1 | 1.00 | reference |  |
| ≤1 | 1.06 | 0.32-3.54 | 0.93 |
| FEV_1_, %pred |  |  |  |
| >50 | 1.00 | reference |  |
| ≤50 | 0.76 | 0.23-2.51 | 0.66 |
| ISWT distance at baseline, m |  |  |  |
| >350 | 1.00 | reference |  |
| ≤350 | 2.65 | 0.75-9.35 | 0.13 |

Odds ratios (ORs) were computed using multivariable logistic regression. ORs > 1 indicate an increased probability of dropping out. BMI = body mass index; COPD = chronic obstructive pulmonary disease; FEV_1_ = forced expiratory volume in 1s; mMRC = modified Medical Research Council dyspnea scale; ISWT = incremental shuttle walking test.

# **Table S9 Adherence to training sessions among participants who completed the 12-week follow-up**

|  | MT  (n=20) | RW  (n=21) | UC  (n=14) |
| --- | --- | --- | --- |
| Mean completion rate, %^§^ | 96.1 ± 7.1 | 97.1 ± 4.3 | - |
| Completion rate < 75% ^\|\|^ | 0 (0) | 0 (0) | - |
| Completion rate ≥ 75% ^\|\|^ | 20 (100) | 21 (100) | - |

^§^ Data are presented as mean (SD). ^||^ Data are presented as N (%). The completion rate of each participant was defined as the percentage of sessions completed (derived using the app data) versus planned. MT = multi-module training; RW = rhythm-guided walking; UC = usual care.

# **Table S10 Satisfaction with the smart app-based PR program among participants who completed the 12-week follow-up**

|  | MT  (n=20) | RW  (n=21) | UC  (n=14) |
| --- | --- | --- | --- |
| Very satisfied | 11 (55%) | 10 (47.6%) | - |
| Satisfied | 9 (45%) | 11 (52.4%) | - |
| Neutral | 0 (0) | 0 (0) | - |
| Dissatisfied | 0 (0) | 0 (0) | - |
| Very dissatisfied | 0 (0) | 0 (0) | - |

# **Table S11 Participants enrolled in each medical center**

|  | N | | |
| --- | --- | --- | --- |
|  | MT  (n=25) | RW  (n=23) | UC  (n=22) |
| China-Japan Friendship Hospital | 7 | 6 | 7 |
| Beijing Tiantan Hospital | 6 | 6 | 5 |
| Beijing Luhe Hospital | 2 | 2 | 1 |
| the Second Affiliated Hospital of Xi’an Jiaotong University | 5 | 5 | 5 |
| Qingdao Municipal Hospital | 5 | 4 | 4 |

MT = multi-module training; RW = rhythm-guided walking; UC = usual care.

# Reference

1. Holland AE, Cox NS, Houchen-Wolloff L, et al. Defining Modern Pulmonary Rehabilitation. An Official American Thoracic Society Workshop Report. Ann Am Thorac Soc 2021; 18: e12-e29.

2. Lewis A, Cave P, Stern M, et al. Singing for Lung Health-a systematic review of the literature and consensus statement. NPJ Prim Care Respir Med 2016; 26: 16080.

3. Rochester CL, Alison JA, Carlin B, et al. Pulmonary Rehabilitation for Adults with Chronic Respiratory Disease: An Official American Thoracic Society Clinical Practice Guideline. Am J Respir Crit Care Med 2023; 208: e7-e26.

4. Singh SJ, Morgan MD, Scott S, et al. Development of a shuttle walking test of disability in patients with chronic airways obstruction. Thorax 1992; 47: 1019-24.

5. Spruit MA, Singh SJ, Garvey C, et al. An official American Thoracic Society/European Respiratory Society statement: key concepts and advances in pulmonary rehabilitation. Am J Respir Crit Care Med 2013; 188: e13-64.

6. Shengyue fenji jiaocheng 1 [Chinese] (a book with a selection of Chinese and international songs from different time periods and genres), ISBN 9787810965255. Beijing: Edition Qiang Long; 2013.

7. Heydon R, Fancourt D, Cohen AJ. The Routledge companion to interdisciplinary studies in singing. Volume III: Wellbeing. Part 1: Singing and Health, 7: Singing for Lung Health (Phone Cave, Adam Lewis, Daisy Fancourt). Routledge; 2020.
